# Isolation and Characterization of Bacteria That Degrade Phosphonates in Marine Dissolved Organic Matter

**DOI:** 10.3389/fmicb.2017.01786

**Published:** 2017-09-26

**Authors:** Oscar A. Sosa, Daniel J. Repeta, Sara Ferrón, Jessica A. Bryant, Daniel R. Mende, David. M. Karl, Edward F. DeLong

**Affiliations:** ^1^Daniel K. Inouye Center for Microbial Oceanography: Research and Education, University of Hawaii, Honolulu, HI, United States; ^2^Department of Oceanography, School of Ocean and Earth Science and Technology, University of Hawaii, Honolulu, HI, United States; ^3^Department of Marine Chemistry and Geochemistry, Woods Hole Oceanographic Institution, Woods Hole, MA, United States

**Keywords:** bacterial degradation, dissolved organic matter (DOM), phosphonate metabolism, C-P lyase, methane, ethylene, oligotrophic conditions

## Abstract

Semi-labile dissolved organic matter (DOM) accumulates in surface waters of the oligotrophic ocean gyres and turns over on seasonal to annual timescales. This reservoir of DOM represents an important source of carbon, energy, and nutrients to marine microbial communities but the identity of the microorganisms and the biochemical pathways underlying the cycling of DOM remain largely uncharacterized. In this study we describe bacteria isolated from the North Pacific Subtropical Gyre (NPSG) near Hawaii that are able to degrade phosphonates associated with high molecular weight dissolved organic matter (HMWDOM), which represents a large fraction of semi-labile DOM. We amended dilution-to-extinction cultures with HMWDOM collected from NPSG surface waters and with purified HMWDOM enriched with polysaccharides bearing alkylphosphonate esters. The HMWDOM-amended cultures were enriched in Roseobacter isolates closely related to *Sulfitobacter* and close relatives of hydrocarbon-degrading bacteria of the *Oceanospirillaceae* family, many of which encoded phosphonate degradation pathways. *Sulfitobacter* cultures encoding C-P lyase were able to catabolize methylphosphonate and 2-hydroxyethylphosphonate, as well as the esters of these phosphonates found in native HMWDOM polysaccharides to acquire phosphorus while producing methane and ethylene, respectively. Conversely, growth of these isolates on HMWDOM polysaccharides as carbon source did not support robust increases in cell yields, suggesting that the constituent carbohydrates in HMWDOM were not readily available to these individual isolates. We postulate that the complete remineralization of HMWDOM polysaccharides requires more complex microbial inter-species interactions. The degradation of phosphonate esters and other common substitutions in marine polysaccharides may be key steps in the turnover of marine DOM.

## Introduction

Microbial heterotrophs in the sunlit region of the ocean are thought to be highly dependent on labile dissolved organic matter (DOM) produced by photosynthetic microorganisms (Cole et al., 1988; Zweifel et al., 1993; Cherrier et al., 1996; Gasol et al., 1998; Kuipers et al., 2000). Recent studies of the transcriptional dynamics of microbial communities in marine surface waters also suggest that the metabolism of the dominant heterotrophic and photoheterotrophic bacterioplankton populations is synchronized to autotrophic processes (Ottesen et al., 2014; Aylward et al., 2015). Despite the tight coupling observed between autotrophic and heterotrophic processes, a fraction of DOM escapes immediate degradation and accumulates in surface waters, turning over slowly as a result of a hypothetical malfunctioning microbial loop wherein DOM cannot be consumed as quickly as it is produced (Thingstad et al., 1997). Some of this “semi-labile” DOM is also exported and degraded below the euphotic zone over seasonal to annual timescales (Hansell et al., 2002; Carlson et al., 2004, 2010), fueling deep sea microbial community respiration (Arístegui et al., 2005).

About one-third of the semi-labile DOM in marine surface waters is HMWDOM that can be collected by ultrafiltration. HMWDOM isolated from surface waters in the NPSG is rich in carbohydrates, approximately 50% of total carbon (C), and its carbohydrate content diminishes rapidly with depth in the upper ocean indicating these are actively consumed by microorganisms (Benner et al., 1992). The spectral properties of HMWDOM are notably similar across ocean basins and freshwater environments suggesting a widespread and common source of this material (Aluwihare et al., 1997; Repeta et al., 2002). HMWDOM carbohydrates consist largely of a family of acylated heteropolysaccharides that have a well conserved monosaccharide composition resembling algal structural polysaccharides (Aluwihare et al., 1997). The radiocarbon value of the monosaccharides from acid-labile carbohydrates in HMWDOM indicates this carbon pool turns over in <3 years (Repeta and Aluwihare, 2006), consistent with the residence time of semi-labile DOM (100–1000 days) estimated by ecosystem modeling approaches (Luo et al., 2010).

Polysaccharides in HMWDOM incorporate large amounts of nitrogen (N) and phosphorus (P) that serve as organic nutrients to support microbial growth. More than half of organic N in HMWDOM occurs as *N*-acetyl amino polysaccharides (Aluwihare et al., 1997, 2005), making this one of the largest N reservoirs in the ocean. HMWDOM polysaccharides also contain phosphate and alkylphosphonate esters, which represent approximately 80% and 20% of the total organic P, respectively (Repeta et al., 2016), and are likely a key source of P to microorganisms. Despite the detailed chemical characterization of HMWDOM, little is known about how microorganisms degrade and metabolize this pool of organic nutrients.

Phosphonates (organophosphorus compounds with a characteristic C-P bond) occur in HMWDOM throughout the water column and across ocean basins (Kolowith et al., 2001). The microbial cycling of HMWDOM polysaccharide phosphonates, namely methylphosphonate (MPn) esters, has recently been shown to be a key pathway by which dissolved methane in the upper ocean reaches concentrations above equilibrium with the atmosphere (Repeta et al., 2016), a phenomenon known as the oceanic methane paradox (Kiene, 1991). Microcosm studies showed that microbial communities from surface waters in the NPSG metabolized MPn and 2-hydroxyethylphosphonate (2-HEP) esters in HMWDOM polysaccharides to obtain P, producing methane and ethylene, respectively, the dealkylation products of these phosphonates (Repeta et al., 2016). Furthermore, a *Pseudomonas stutzeri* bacterium isolated from HMWDOM-amended seawater was shown to employ the C-P lyase pathway (Metcalf and Wanner, 1993a,b; White and Metcalf, 2004) to breakdown the MPn and 2-HEP esters in HMWDOM polysaccharides in response to P-limitation (Repeta et al., 2016). These findings supported the role of HMWDOM as a key source of organic P to microbial communities and highlight its utility as a model substrate to study microbial DOM cycling.

In this study we identify and characterize populations of organisms from low primary productivity regions in the NPSG capable of degrading constituents of semi-labile DOM. Seawater samples collected at the bottom of the euphotic zone near the deep chlorophyll maximum (DCM) and from the upper mesopelagic zone were supplemented with HMWDOM isolated from surface water which stimulated the growth of select bacterial populations. The growth characteristics and predicted metabolic functions of the bacterial isolates identified provide information of the bio-availability and cycling of major chemical constituents of HMWDOM. Results of this study demonstrate that phosphonates associated with HMWDOM carbohydrates are readily available to bacteria but also suggest that inter-species microbial interactions are required to fully degrade these carbohydrates. Our observations also link widespread bacterial clades like Roseobacter to semi-labile DOM cycling and to the aerobic production of methane in the ocean.

## Materials and Methods

### Collection and Characterization of HMWDOM and Purification of the Polysaccharide-Rich Fraction

HMWDOM with a nominal molecular weight >1 kDa was collected by ultrafiltration of Hawaii seawater pumped from offshore through a 20 m-depth intake pipe at the National Energy Laboratory Hawaii Authority in Kona, HI, United States in February, 2013. HMWDOM ultrafiltration, chromatographic purification of polysaccharide-rich fractions, and chemical and spectroscopic analyses were performed as described previously (Repeta et al., 2016). The P content of purified HMWDOM polysaccharides was determined by the total dissolved P method described by Karl and Björkman (2001) which is based on the high temperature wet persulfate oxidation method for seawater (Menzel and Corwin, 1965) followed by phosphate determination by the magnesium-induced coprecipitation and standard Murphy-Riley molybdenum blue reaction.

### Preparation of Dilution-to-Extinction Cultures Amended with HMWDOM

Seawater samples for culturing experiments were collected at the field site of the Hawaii Ocean Time-series program, Station ALOHA, a region representative of the general conditions of the NPSG (Karl and Lukas, 1996). Seawater from the DCM (95 m) and from the top of the mesopelagic zone (250 m) was sampled with 12 L Niskin bottles attached to a Sea-Bird CTD-rosette during the Hawaii Ocean Experiment Budget of Energy I cruise on March 23, 2014^1^. The culturing strategy was based on a modified high-throughput dilution-to-extinction culturing method (Connon and Giovannoni, 2002) in which samples are amended with HMWDOM collected by ultrafiltration (Sosa et al., 2015). Total cell abundances in the seawater samples collected were measured at sea with an Influx flow cytometer (BD Biosciences, San Jose, CA, United States). Briefly, triplicate 1 mL sub-samples were fixed with 15 μL of 16% paraformaldehyde for 10 min and stained with 5 μL of a 200x solution of SYBR^®^ Green I (Invitrogen, Carlsbad, CA, United States) for 20 min in darkness. Total cell concentrations from each sample were used to calculate the dilutions necessary to obtain a final concentration of 2–3 cells mL^-1^ for dilution-to-extinction. Seawater samples were diluted with sterile seawater from 125 m depth obtained by tangential flow filtration as described previously (Sosa et al., 2015). The seawater for this medium was collected at Station ALOHA during the Hawaii Ocean Time-series cruise no. 261 in March 2014 prior to the culturing experiments. The total organic carbon (TOC) content of this medium was 67 μM, as determined by high-temperature combustion using a V series Shimadzu TOC analyzer (Shimadzu Scientific Instruments, Columbia, MD, United States). Control dilution-to-extinction samples were prepared with this medium without HMWDOM additions (treatment I). To prepare dilution-to-extinction samples with HMWDOM (treatments II–IV), freeze-dried HMWDOM was first dissolved in ultrapure water and filter-sterilized through a 0.22 μm Supor membrane syringe filter (Pall Corp., Port Washington, NY, United States). Approximately 7–18 mg of HMWDOM (2–5 mg C) was subsequently added to 1 L dilution-to-extinction samples resulting in TOC concentrations ranging from 162 to 405 μM. Because the HMWDOM samples are rich in C relative to N and P, the medium was also amended with inorganic N (60 μM NaNO_3_ and 4 μM NH_4_Cl) and P (7 μM H_3_PO_4_). This approximated the final C, N, and P content of HMWDOM-amended media to that of marine bacterial biomass (C:N:P ∼45:9:1; Goldman et al., 1987). Each dilution-to-extinction sample was inoculated into 720 wells (1 mL per well) in non-tissue culture treated 48-well cultivation plates (Corning, Inc., Corning, NY, United States). Sterile dilution medium, with and without HMWDOM, was also incubated in cultivation plates to evaluate the presence of microbial contamination in the base seawater medium and in HMWDOM. DCM samples were incubated at 26°C under a diurnal cycle consisting of a 12 h period of continuous light (30 μmol m^-2^ s^-1^ photons of photosynthetically active radiation) and a 12 h dark period. Mesopelagic samples were incubated at 25°C in darkness. All cultivation plates were incubated for 10–11 weeks before evaluating culture growth.

### Growth Screen of Dilution-to-Extinction Cultures

To evaluate the effect of HMWDOM additions on the growth of dilution-to-extinction cultures, 100 μl were sub-sampled from each well to enumerate cells stained with SYBR^®^ Green I on a GUAVA easyCyte PLUS flow cytometer (Millipore). Flow cytometry data were analyzed with the flowCore and prada R packages as described previously (Sosa et al., 2015). Wells with densities >1 × 10^4^ cells mL^-1^ were considered to test positive for growth. Cultures identified in positive wells were maintained by transferring samples into 10 mL of the same sterile dilution-to-extinction medium in which each was isolated, including the same concentration of HMWDOM and inorganic nutrients.

### DNA Purification and Whole Genome Shotgun Sequencing

To obtain genomic DNA for whole genome shotgun sequencing, cultures that remained viable after transferring were grown in 100 mL of the same seawater medium used for dilution-to-extinction without added HMWDOM. Cells were harvested on 0.22 μm Supor membrane disc filters (Pall Corp.) and stored at -80°C in sucrose-based lysis buffer (40 mM EDTA, 50 mM Tris [pH 8.3], and 0.75 M sucrose). Cells were incubated in lysis buffer containing 2 mg mL^-1^ of lysozyme (Sigma–Aldrich, St. Louis, MO, United States) for 30 min at 37°C and subsequently with 0.8 mg mL^-1^ of proteinase K (Roche, Basel, Switzerland) and 1% SDS for 2 h at 55°C. DNA was purified from the lysate using a chemagic Magnetic Separation Module I (Perkin Elmer, Waltham, MA, United States). Purified DNA samples were quantified using the PicoGreen^®^ assay (Invitrogen) and the DNA concentrations were normalized before sequencing. DNA samples were prepared for sequencing using a Nextera XT 96 DNA sample preparation kit (Illumina, San Diego, CA, United States) to obtain indexed paired-end libraries. Libraries were sequenced with a 2 × 300 nt paired-end MiSeq run (Illumina).

### Genome Assembly and Annotation

For cultures with high quality Illumina data, paired-end reads were first assembled using MIRA version 4.9.3 (Chevreux et al., 1999) with options for removing remaining Illumina adapters, low quality stretches, phiX174 standard, as well as other common Illumina artifacts. This generated a very conservative assembly which was used to predict genes. As part of the MIRA assembly process, a set of cleaned/clipped reads was also generated. This set of clean reads was used as input to SPAdes version 3.5 (Bankevich et al., 2012) with default parameters and explored with different *k*-mer settings. The resulting contigs were annotated through NCBI’s Prokaryotic Genome Annotation Pipeline (Tatusova et al., 2013).

For the remaining cultures, FastQ files from the MiSeq run were imported into the CLC Genomics Workbench (CLC bio, Aarhus, Denmark). Reads were assembled into contigs using CLC’s *de novo* assembler with automatic word and bubble sizes, a minimum contig length of 200, insertion and deletion costs set to 3, mismatch cost set to 2, length fraction set to 0.5, and the similarity fraction set to 0.8. These contigs were used to identify isolates with limited Illumina data.

### Phylogenetic Placement of Cultures

For cultures with high-quality Illumina data, full-length small subunit (SSU) rRNA sequences were obtained from the annotated assemblies. For the remaining cultures, full-length or partial SSU rRNA sequences were extracted manually from contigs ≥1000 nt in length assembled in the CLC genomics workbench. To identify SSU rRNA sequences in these contigs, blastn was implemented in the CLC genomics workbench with default parameters (number of threads = 1, filter low complexity = yes, mask lower case = no, expectation value = 10, word size = 11, match = 2, mismatch = -3, gap costs: existence = 5 and extension = 2, and maximum number of hits = 100) using the SILVA 115 NR99 database. Complete and near full-length SSU rRNA genes identified in the assembled data were aligned using the SILVA Incremental Aligner (SINA) version 1.2.11 and classified with the least common ancestor method (Pruesse et al., 2012). The alignment was imported into ARB (Ludwig et al., 2004) and merged with the SILVA “All-Species Living Tree” (LTPs115) SSU reference tree (Munoz et al., 2011). A total of 240 sequences including closely related reference sequences from SILVA were included for phylogenetic comparisons. The sequence alignment was filtered in ARB to exclude gaps, missing data, ambiguous nucleotide positions, and lower case positions. The filtered alignment contained 1172 comparable nucleotide positions across all sequences. Phylogenetic analysis was implemented using the Neighbor-Joining method (Saitou and Nei, 1987) with 1000-replicate bootstrap confidence limits (Felsenstein, 1985) in MEGA6 (Tamura et al., 2013). The evolutionary distances were computed using the Kimura 2-parameter method (Kimura, 1980) and are in the units of the number of base substitutions per site. The rate variation among sites was modeled with a gamma distribution (shape parameter = 2).

### Functional Analysis of Coding DNA Sequences

We explored the functional annotations of protein coding DNA sequences (CDS) to assess the potential of our isolates to degrade known chemical constituents of HMWDOM, namely carbohydrates and phosphonates. To determine the potential of carbohydrate degradation, CDSs were annotated with the Carbohydrate-Active EnZyme (CAZy) database (Cantarel et al., 2009) using HMMER3.0 (Eddy, 2009) and hidden Markov models of CAZy signature domains (Yin et al., 2012). We compared the proportion of CDSs with predicted CAZy domains in each of the 55 assembled genomes to those of well-known bacteria specialized in carbohydrate degradation. The analysis also targeted protein sequences associated with the C-P lyase pathway for alkylphosphonate degradation (Metcalf and Wanner, 1993a,b). BLASTp was employed to identify best matching C-P lyase pathway operons present in the genomes of HMWDOM isolates using as queries the protein sequences of C-P lyase pathway operons identified by Martínez et al. (2013) in the NPSG (Supplementary Table 1). In addition, the NCBI genome annotations of CDSs of these isolates were explored to identify additional proteins involved in phosphonate metabolism.

### Gas Chromatography Analysis of Bacterial Cultures Amended with HWMDOM Polysaccharides or Phosphonates

Cultures of *Sulfitobacter* sp. HI0054 were amended with phosphonates or purified HMWDOM polysaccharides to test and measure the production of hydrocarbon gas products expected from dealkylation of phosphonates. *Sulfitobacter* cultures were pre-grown in seawater medium containing glycerol (0.2 mM C) and ammonium (0.8 mM). To force cells to take up phosphate present in natural seawater, P was not added to the medium. The medium was also supplemented with PRO99 medium trace metals (Moore et al., 2007) and with a vitamin cocktail based on the RMP medium for the cultivation of cyanobacteria (Supplementary Table 2). The culture was grown for 3 days at 25°C, stirred gently, and bubbled with filtered air to replenish oxygen. Subsequently, culture samples were aliquoted into 72 mL serum vials to prepare incubations with phosphonates or HMWDOM as follows: (1) control samples with no P added; (2) HMWDOM polysaccharides (27 μg mL^-1^) containing approximately 855 μM C and 3.9 μM P; (3) methylphosphonate (MPn, 250 nM); (4) 2-hydroxyethylphosphonate (2-HEP, 250 nM); and (5) ethylphosphonate (EPn, 250 nM). Based on the C:P ratio of HMWDOM polysaccharides and the proportion of total P comprised of phosphonates (20%; Repeta et al., 2016), the HMWDOM amendment was estimated to contain ∼780 nM phosphonates. Triplicate samples for each treatment were crimp-sealed with butyl/PTFE-faced plugs (Wheaton, Millville, NJ, United States) and aluminum collars and incubated at 25°C for 3 days.

Dissolved methane, ethylene, and ethane concentrations were measured by gas chromatography using a gas stripping and cryo-trap concentration method similar to that of Tilbrook and Karl (1995), and described in Repeta et al. (2016). Dissolved gases were purged from a known volume of culture with ultra high purity helium. The volume of sample purged, determined by weight, was approximately 50 mL. During the gas stripping procedure, the gas stream passed through Drierite^®^ and Ascarite^®^ columns to remove water vapor and carbon dioxide, and then the gases were concentrated in a 80–100 mesh Porapak-Q^®^ trap cooled in liquid N_2_. Once the gas extraction was completed (12 min), the trap was heated with boiling water and the concentrated gases were released and injected into the gas chromatograph (model 7980A, Agilent Technologies, Inc., Santa Clara, CA, United States). The gases were separated in a GS-CarbonPLOT capillary column (30 m × 0.320 mm I.D., 3.0 μM; Agilent Technologies, Inc.) and analyzed with a flame ionization detector (FID). The FID was calibrated by injecting different size loops of a gas mixture standard containing 10 ppm of methane, ethylene, and ethane in pure N_2_ (Scott-Marrin, Inc., Riverside, CA, United States). The loops were injected into the purging chamber and concentrated into the Porapak-Q^®^ trap following the same procedure as for the seawater samples.

### Environmental Representation of C-P Lyase in the NPSG

Metagenomic coverage of SSU rRNA gene sequences of bacteria isolated from HMWDOM-amended treatments encoding C-P lyase was used to estimate their abundance throughout the water column at Station ALOHA. The metagenomes interrogated were obtained from seawater samples collected during 12 Hawaii Ocean Time-series cruises between August 2010 and December 2011 and correspond to the datasets comprising the ALOHA gene catalog (Mende et al., 2017). The SSU rRNA sequences of the bacterial isolates were assigned to operational taxonomic units (OTUs) by first combining them with a collection of sequences from the SILVA database representing OTUs (97% sequence identity) developed for QIIME (Caporaso et al., 2010; Quast et al., 2013; SILVA release 128). The combined sequences were then clustered into OTUs (97% sequence identity) using vsearch (Rognes et al., 2016) and a representative sequence from each OTU was selected. The normalized abundances of OTUs representing our isolates were estimated by mapping SSU rRNA gene fragments extracted from Station ALOHA shotgun metagenomes to the OTU reference sequences using bowtie2 (Langmead and Salzberg, 2012). Fragments were assigned to the OTU with the best alignment provided there was greater than 97% sequence identity over at least 70 bps. The number of gene fragments mapping to these OTUs was then divided by the total number of mapped fragments.

The ALOHA gene catalog (Mende et al., 2017) further provides normalized abundances for each gene and functional group as estimates of copy number per average genome size for each metagenome. To estimate the abundance of C-P lyase pathway genes at Station ALOHA, the individual genes in the C-P lyase operons of the HMWDOM isolates were first assigned to eggNOG orthologous groups (OG) (Huerta-Cepas et al., 2016) (PhnG: COG3624, PhnH: COG3625, PhnI: COG3626, PhnJ: COG3627, PhnK: COG4107, PhnL: COG4778, PhnM: COG3454, PhnN: ENOG4111MI2 and COG3709). Subsequently, the abundances of these OGs were obtained from the ALOHA gene catalog and mean abundances were plotted by sampling depth to explore the importance of this pathway throughout the water column. Differences in mean abundances between depths were evaluated using a Tukey multiple comparisons method adjusted for multiplicity (Bonferroni correction) implemented with R package multcomp.

### HMWDOM Dose Growth Response Tests

To determine if HMWDOM supported increased cell yields of bacterial isolates relative to unamended seawater, we grew cultures with increasing concentrations of HMWDOM or purified HMWDOM polysaccharides. Sterile seawater media used for dilution-to-extinction (including the same inorganic nutrient additions) was amended with PRO99 trace metals (Moore et al., 2007) and a vitamin cocktail based on the RMP medium for the cultivation of cyanobacteria (Supplementary Table 2). In this experiment, cultures of two representative isolates were supplemented with 20, 40, or 60 μg mL^-1^ of HMWDOM polysaccharides. In a second experiment, additional cultures were amended with 7.2, 14.4, or 21.6 μg mL^-1^ of HMWDOM or with 14.4 or 28.8 μg mL^-1^ of purified HMWDOM polysaccharides. The control media consisted of seawater without HMWDOM amendments. All tests were conducted in triplicate. Samples were inoculated with cultures maintained in seawater media without HMWDOM amendments at 25°C in darkness. Growth was measured over the course of a week as total SYBR^®^ Green I-stained cells on a GUAVA easyCyte PLUS (Millipore) flow cytometer. To evaluate if HMWDOM treatments had a significant positive effect on growth, the average cell yields over stationary phase were compared between HMWDOM treatments and unamended seawater using a one-tailed Dunnett test implemented in R with package multcomp.

### Carbon Substrate Utilization Profiles of Bacterial Isolates

To characterize the types of carbon compounds metabolized by bacterial isolates, representative isolates were assayed for increased cell yields in Biolog multi-carbon plates (PM1 and PM2A) containing a total of 190 different organic compounds (Biolog, Hayward, CA, United States). The exact formulation of Biolog plates is proprietary but is similar to the recipes published by Bochner et al. (2001) which utilize 25 mM carbon compound concentrations and 2 and 5 mM inorganic P and N concentrations, respectively. In our experiments, each compound was dissolved in 100 μL of sterile water and treated as a 10,000-fold concentrated stock. The seawater medium was also amended with PRO99 trace metals and RMP medium vitamins (Supplementary Table 2). Bacterial cultures were grown in 48-well cultivation plates (Corning, Inc.). The Biolog compounds were added to a final one-fold concentration. Samples were incubated for 3–7 days at 26°C. To determine which compounds supported growth, cell concentrations were obtained by flow cytometry several times during the incubation period from 100 μl sub-samples stained with SYBR^®^ Green I.

The cell yields at each time point were analyzed using a modified outlier identifier method (Leys et al., 2013) which relies on the median absolute deviation (MAD) to determine upper (median + 2 × MAD) and lower (median - 2 × MAD) confidence intervals of the data. MAD was implemented in R with function MAD and default options. Substrates deemed to support growth had cell yields greater than the upper MAD-based thresholds. In addition, the relative growth effect of each Biolog compound was evaluated by normalizing the cell yield by the median cell yield across all test compounds (190 total) and log_2_-transforming this ratio to produce a doubling scale (log_2_ fold-change). MAD-based positive growth agreed well with cell yields resulting in a log_2_ fold change >1 and negative growth with log_2_ fold-change <-1. The median was selected to analyze the data and normalize cell yields because its value closely approximated the cell yields in cultures with no Biolog carbon substrate added and because most test substrates did not result in considerable growth.

## Results

### Elemental and Chemical Composition of HMWDOM

HMWDOM collected from Hawaii surface waters was composed of 30% carbon by weight. The polysaccharide chromatographic fraction was composed of 38–40% carbon by weight. The C:N ratio of these samples were similar, 12.6 and 12.9, respectively. HMWDOM polysaccharides were composed of 0.47% P by weight with a C:P ratio of 220. ^31^P-NMR analysis indicated the polysaccharide fraction consisted of phosphonate (20% total P), phosphate (70% of total P) and pyrophosphate (10% of total P) esters as reported previously (Repeta et al., 2016). The chromatographic separation procedure removed organic constituents consistent with the chemical spectra of humic substances and produced fractions consisting of ≥90% polysaccharides.

### HMWDOM Amendments and Recovery of Cultures

Mean (±standard deviation, *n* = 3) cell abundances in DCM and mesopelagic seawater samples used for dilution-to-extinction culturing were 5.0 × 10^5^ ± 1.4 × 10^4^ cells mL^-1^ and 1.1 × 10^5^ ± 2.6 × 10^3^ cells mL^-1^, respectively. These cell densities are characteristic of the NPSG near Station ALOHA (Campbell et al., 1997). HMWDOM amendments to dilution-to-extinction cultures increased the total organic carbon (TOC) concentration of the cultivation media 3–7 times (230–472 μM) over the TOC of the control medium (67 μM) and the TOC values typically measured in surface waters at Station ALOHA (Supplementary Table 3).

**FIGURE 1:**
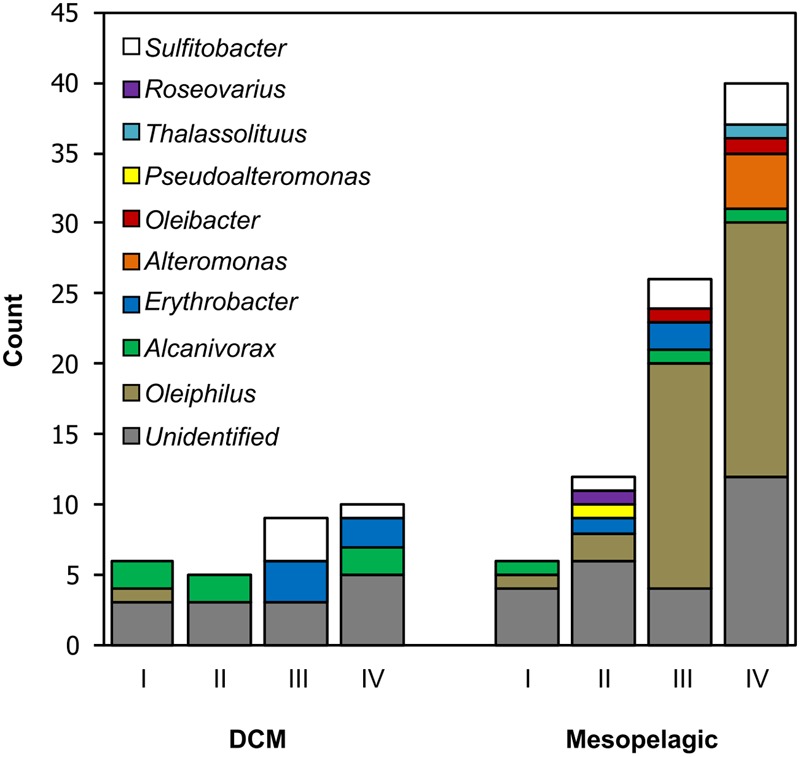
Number and identity of bacterial isolates obtained in dilution-to-extinction culturing experiments. Column I represents control samples lacking HMWDOM amendments and columns II–IV indicate samples with HMWDOM amendments: treatment II: 7.2 mg L^-1^; treatment III: 18 mg L^-1^; treatment IV: 7.2 mg L^-1^ (purified HMWDOM polysaccharides). The column height indicates the number of isolates that were viable and sub-cultured and the column color code indicates the SSU rRNA gene-based identification of isolates. Unidentified samples were positive wells that did not remain viable after sub-culturing and could not be sequenced. The total column height indicates the number of positive wells (>1 × 10^4^ cells mL^-1^) originally detected in the flow cytometry growth screen.

The distribution of cell densities in dilution-to-extinction samples and the number of wells that tested positive for growth in each control sample or HMWDOM treatment during the flow cytometry screen are presented in Supplementary Table 4. The difference in the proportion of positive wells recovered from DCM samples was not significant between treatments, *X*^2^(3, *N* = 30) = 2.27, *p* > 0.05. In contrast, there was a significant difference in the number of positive wells recovered between treatments in mesopelagic samples, *X*^2^(3, *N* = 84) = 32.95, *p* < 0.05. Notably, HMWDOM amendments to mesopelagic samples (treatments II–IV, **Figure 1**) recovered 2–8 times more positive wells than the control experiment (treatment I, **Figure 1**). A total of 74 cultures out of 114 positive wells, 16 from the DCM and 58 from the mesopelagic, remained viable after subsequent transfers to HMWDOM-amended media and were grown for whole genome shotgun sequencing.

### Genome Assemblies and Phylogeny of Cultures

Whole genome shotgun sequencing generated Illumina data for all 74 cultures of which 55 resulted in high-quality assemblies. The raw Illumina data was deposited in the NCBI sequence read archive under BioProject PRJNA305749 and is available under accessions SRX2766079–SRX2766152. The annotated assemblies were deposited in the DNA Data Bank of Japan, the European Nucleotide Archive, and GenBank and are available under accessions LWEI00000000–LWGK00000000. Individual isolate identifications, accession numbers, genome assembly metrics, and gene composition are summarized in Supplementary Tables 5 and 6.

Small subunit rRNA sequences of all 74 cultures fell under the classification of gammaproteobacteria (Supplementary Figure 1) or alphaproteobacteria (Supplementary Figure 2). The identity of SSU rRNA sequences in the SILVA database (119) best matching our bacterial isolates is presented in Supplementary Table 7. In addition to full-length SSU rRNA sequences, several partial SSU rRNA sequences were identified in the genome assemblies. In all but two of these assemblies, the partial sequences were 99–100% similar to the full-length SSU rRNA sequences (Supplementary Table 5).

The number of isolates from each bacterial clade identified in the different treatments is summarized in **Figure 1**. The most common bacterial group isolated was closely related to the gammaproteobacterium *Oleiphilus messinensis* (Golyshin et al., 2002), enriched particularly in treatments III and IV in mesopelagic samples. The Roseobacter subclade *Sulfitobacter* was the second most common group in both DCM and mesopelagic samples amended with HMWDOM. Similarly, the *Erythrobacter* isolates were present in samples from both depths and only in treatments with HMWDOM. In turn, the *Alteromonas* isolates only occurred in mesopelagic samples amended with HMWDOM polysaccharides (treatment IV). In contrast, the *Alcanivorax* isolates were identified in all treatments and in samples from both depths, including unamended samples (treatment I). The remaining isolates were present in at least one HMWDOM treatment and were absent from the control treatment (I).

### Phosphonate Degradation Pathways in Bacterial Isolates

**FIGURE 2:**
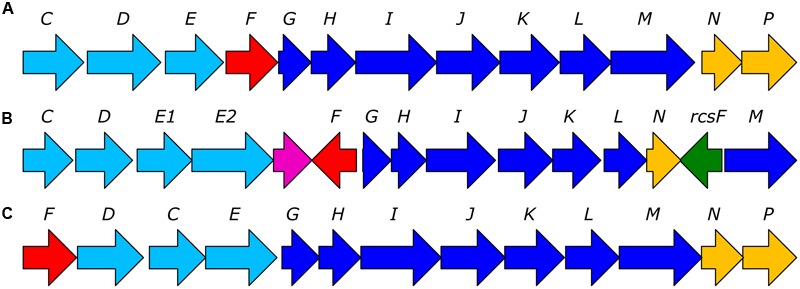
Representative bacterial C-P lyase operons identified in HMWDOM enriched bacterial isolates. **(A)** The C-P lyase operon of *Pseudomonas stutzeri* HI00D1 (Repeta et al., 2016). **(B)** The C-P lyase operon of *Sulfitobacter* sp. HI0054. **(C)** A hypothetical C-P lyase operon identified in *Oleiphilus* sp. HI0066. The arrow color indicates genes involved in phosphonate transport (light blue), transcriptional regulation (red), catalytic functions (blue), auxiliary functions (yellow), and undetermined functions (green and violet).

Surveying the functional assignments of CDSs revealed the presence of complete C-P lyase pathway operons in 10 out of 55 genomes analyzed. *Sulfitobacter* sps. HI0021, HI0027, HI0054, HI0076, and HI0082, as well as *Roseovarius* sp. HI0049 encoded C-P lyase operons (**Figure 2B**) that were most similar to the *Rhodobacteraceae* operon B3TF_MPn_2 identified by Martínez et al. (2013) in surface waters near Hawaii (Supplementary Table 1). The *Roseovarius* sp. HI0049 draft genome encoded all predicted C-P lyase genes required for phosphonate degradation and additional putative auxiliary functions. These genes included a tetR family transcriptional regulator, phosphonate ABC transporters *phnCDE1E2*, a putative chloramphenicol acetyltransferase matching the transferase in the B3TF_MPn2 cluster, and a homolog of *phnM1* (contig LWFA01002944), *phnFGHIJK* (contig LWFA01001941) and predicted protein homologs to *phnM, rcsF* (a phosphoesterase perhaps analogous to *phnP*; Martínez et al., 2013), and *phnNL* (contig LWFA01002897). The C-P lyase operons of *Sulfitobacter* isolates HI0021 (contig LWEP01000037), HI0027 (contig LWER01000183), *Sulfitobacter* sp. HI0054 (contig LWFD01000024), HI0076 (contig LWFQ01000564), and of HI0082 (contig LWFW01000191) were highly similar to the B3TF_MPn_2 operon but lacked a copy of *phnM1*. Also lacking in all but *Sulfitobacter* sp. HI0054 was a putative transferase located between *phnE2* and *phnM1* in the *Rhodobacterales* B3TF_MPn_2 cluster. Four phylogenetically similar *Oleiphilus* isolates (HI0009, HI0066, HI0067, and HI0125, Supplementary Figure 1) also encoded putative C-P lyase pathway operons. The putative C-P lyase operons of the *Oleiphilus* isolates shared 29–52% nucleotide sequence identity to gammaproteobacterium IMCC1989 B3TF_MPn_1 operon identified by Martínez et al. (2013) and contained all the genes in the *P. stutzeri* HI00D01 C-P lyase operon (**Figure 2A**). The C-P lyase gene clusters of isolates HI0009 and HI0066 were predicted to comprise of *phnFDCEGHIJKLMNP* (**Figure 2C**). The two additional *Oleiphilus* isolates (HI0067 and HI0125) encoded all these genes but the assemblies could not resolve them together in the same contig.

In addition to C-P lyase, these same *Oleiphilus* isolates encoded genes bearing high similarity to *phnW* and *phnX*, which encode 2-aminoethylphosphonate:pyruvate aminotransferase and phosphonoacetaldehyde hydrolase, respectively. The former converts 2-aminoethylphosphonate into phosphonoacetaldehyde and alanine and the latter cleaves phosphonoacetaldehyde into acetaldehyde and phosphate (Quinn et al., 2007). *Pseudoalteromonas shioyasakiensis* HI0053 and *Thalassolituus* sp. HI0120 also carry genes annotated as *phnW* and *phnX*. *Roseovarius* sp. HI0049 carries gene *phnY*, which encodes phosphonoacetaldehyde dehydrogenase, an enzyme that mediates the formation of phosphonoacetate (Agarwal et al., 2014).

### Degradation of Phosphonates Associated with HMWDOM Polysaccharides

The ability of *Sulfitobacter* sp. HI0054 to degrade alkylphosphonates and phosphonates associated with HMWDOM polysaccharides was tested using batch cultures incubated in gas tight vials. Culture samples amended with MPn, 2-HEP, or ethylphosphonate (EPn) accumulated dissolved methane, ethylene, and ethane, respectively (**Table 1**). Based on the average gas concentrations measured in these cultures, an estimated 87–93% of the total phosphonate added (250 nM) was degraded during the incubation period. *Sulfitobacter* cultures grown with HMWDOM polysaccharides as sole P source produced methane and ethylene and reached cell yields comparable to samples amended with alkylphosphonates (**Table 1**).

**Table 1:** Hydrocarbon gas production and cell yields of *Sulfitobacter* sp. HI0054 cultures amended with HMWDOM polysaccharides or phosphonates.

Treatment	Methane (nM)	Ethylene (nM)	Ethane (nM)	Cells mL^-1^
No phosphorus	**-**0.2 ± 0.0	0.1 ± 0.0	0.0 ± 0.0	2.6E+07 (3%)
HMWDOM polysaccharides	6.0 ± 0.1	11.1 ± 0.0	0.1 ± 0.0	3.9E+07 (8%)
Methylphosphonate	217.9 ± 3.2	0.1 ± 0.0	0.0 ± 0.0	3.9E+07 (3%)
2-Hydroxyethylphosphonate	**-**0.1 ± 0.1	232.9 ± 0.0	0.0 ± 0.0	3.5E+07 (3%)
Ethylphosphonate	**-**0.2 ± 0.0	0.1 ± 0.0	217.6 ± 4.6	3.8E+07 (5%)

*Indicated are the mean and standard deviations of the final gas concentrations in the samples minus the gas concentration in the media at the beginning of the incubation. Also indicated are the cell concentrations (mean and coefficient of variation) of the samples at the end of the incubation.*

### Abundance of HMWDOM-Enriched Bacterial Isolates and of C-P Lyase Pathway Genes in the NPSG

**FIGURE 3:**
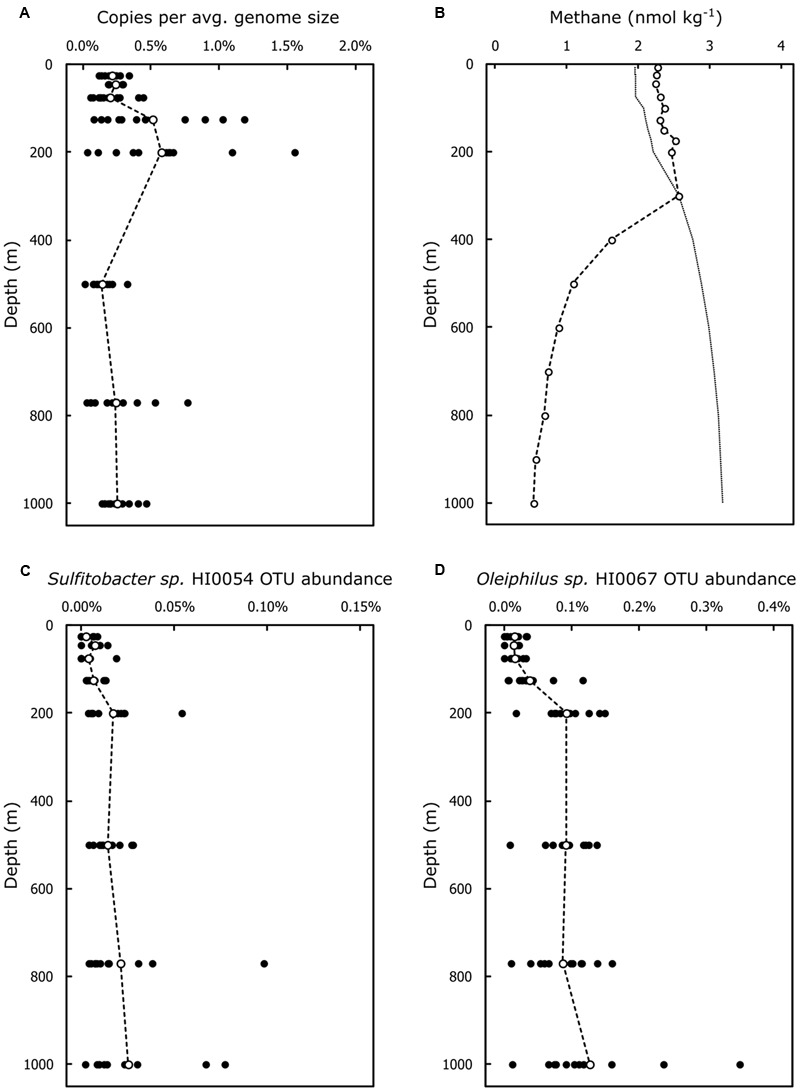
Abundance of the C-P lyase pathway genes and of OTUs representative of HMWDOM enriched bacterial isolates encoding C-P lyase at Station ALOHA. **(A)** The average copy number of C-P lyase pathways genes with catalytic functions normalized to average genome size. **(B)** Typical depth profile of methane concentrations in the water column at Station ALOHA. Indicated are the average methane concentrations in samples collected during Spring 2013 (Hawaii Ocean Time-series cruises 249–251). The predicted methane concentrations in equilibrium with the atmosphere are indicated by the dotted line. **(C)** OTU proportional abundance of SSU rRNA sequences representative of *Sulfitobacter* sp. HI0054. **(D)** OTU proportional abundance of SSU rRNA sequences representative of *Oleiphilus* sp. HI0067. Closed circles represent abundances for individual samples. Open circles indicate the mean abundances for all samples at a given depth.

A survey of the metagenomic ALOHA gene catalog representative of the open ocean near Hawaii (Mende et al., 2017) revealed the presence of C-P lyase pathway genes throughout the water column, on average 1 copy in every 300–400 genomes (**Figure 3A**). C-P lyase pathway genes encoding catalytic functions peaked at 125 and 200 m depths, respectively. At these depths roughly 1 in every 200 genomes (0.5%) encoded a C-P lyase gene. The mean abundance at 125 m depth was only significantly different from the abundance at 500 m (Bonferroni adjusted *p* = 0.01625). Mean C-P lyase abundance at 200 m depth was significantly different from the abundances at 25, 75, 500, and 770 m depths (Bonferroni adjusted *p* < 0.05). The sample to sample variability of C-P lyase gene abundance was also greatest at 125 and 200 m depths relative to other depths sampled (**Figure 3A**).

The abundance estimates of OTUs representative of our bacterial isolates, including those encoding C-P lyase gene operons, are presented in Supplementary Table 8. The OTUs representative of our bacterial isolates comprised on average 0.08% of the community throughout the water column and never exceeded 1%. The *Sulfitobacter* isolates with C-P lyase were mapped to two different rRNA OTUs. One included *Sulfitobacter* sps. HI0021, HI0027, and HI0054 and contributed approximately to 0.005% of the community in the upper 125 m of the water column and 0.01% below 200 m (**Figure 3C**). The remaining *Sulfitobacter* isolates with C-P lyase (HI0076 and HI0082) mapped to another OTU that made up on average 0.23% of the community throughout the water column (Supplementary Table 8). The *Oleiphilus* isolates encoding a C-P lyase-like operon (**Figure 2C**) were also found in two OTUs. The OTU that included *Oleiphilus* sp. HI0067 comprised an average of 0.13% of the community throughout the water column, and showed a marked increase in abundance below 200 m (**Figure 3D**). The second OTU including the C-P lyase-containing *Oleiphilus* isolates had an estimated abundance close to 0.007% consistently throughout the water column.

### Isolate Growth Response to HMWDOM

**FIGURE 4:**
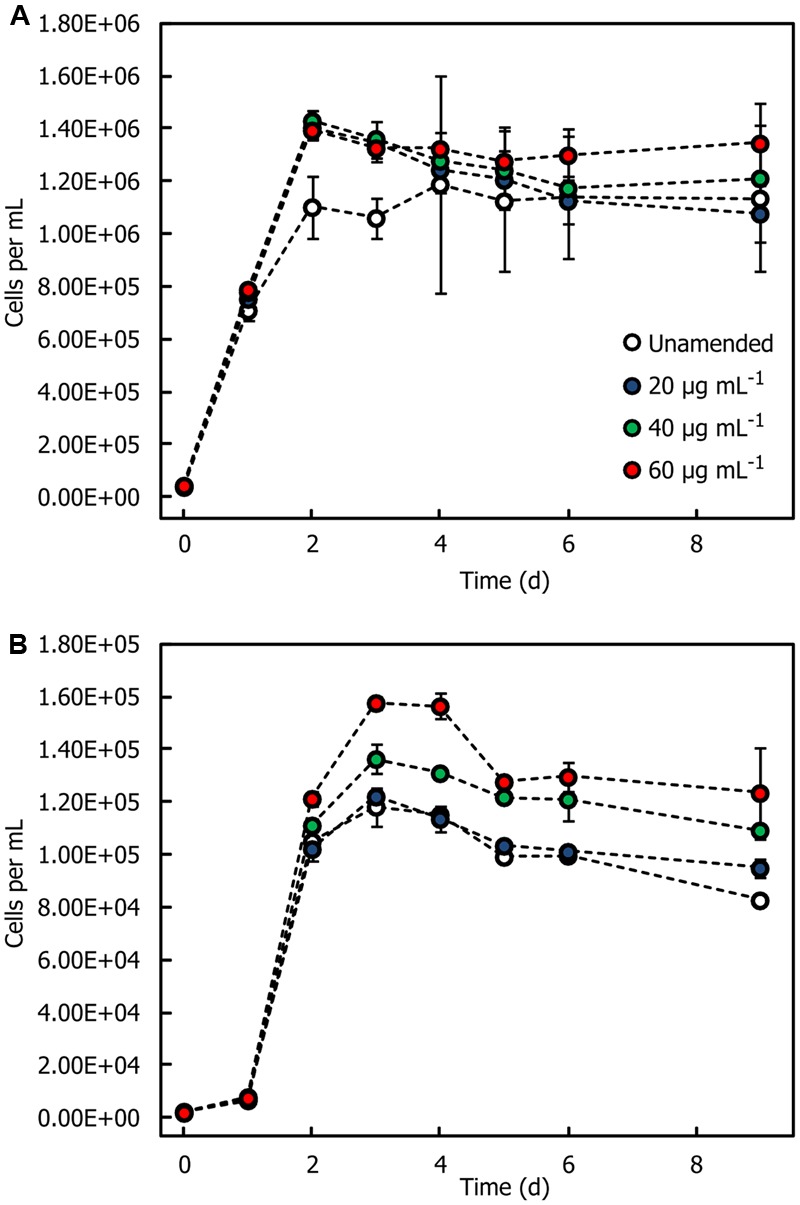
Growth response of bacterial isolates in seawater amended with HMWDOM polysaccharides. **(A)** *Sulfitobacter* sp. HI0054. **(B)** *Oleiphilus* sp. HI0066. Treatments correspond to cultures grown in unamended seawater that had been enriched in nutrients, vitamins and trace metals, and seawater amended with 20, 40, and 60 μg mL^-1^ of HMWDOM polysaccharides, respectively. The plot indicates the mean cell count of triplicate cultures. Error bars represent one standard deviation.

Two representative isolates, *Sulfitobacter* sp. HI0054 and *Oleiphilus* sp. HI0066, were grown in a seawater medium supplemented with trace metals and vitamins and with different concentrations of HMWDOM polysaccharides. Both isolates showed small increases in cell concentrations in media amended with HMWDOM polysaccharide relative to unamended seawater (**Figure 4**). In the case of *Sulfitobacter* sp. HI0054, HMWDOM additions of 40 and 60 μg mL^-1^ increased cell yields by 5.0–9.5 × 10^4^ cells mL^-1^ relative to unamended seawater (Bonferroni-adjusted *p* = [0.0063, 0.0008]). For *Oleiphilus* sp. HI0066 cultures, HMWDOM additions of 40 and 60 μg mL^-1^ stimulated cell growth by 2 × 10^3^ to 1.7 × 10^4^ cells mL^-1^ over unamended seawater (Bonferroni-adjusted *p* = [0.0368, 0.0009]). HMWDOM additions of 20 μg mL^-1^ were not sufficient to induce significant increases in cell yields (Bonferroni-adjusted *p* > 0.05). *P. shioyasakiensis* HI0053, *Erythrobacter* sp. HI0063 (not shown), *Erythrobacter* sp. HI0077, and *Oleibacter* sp. HI0113 *Alcanivorax* sp. HI0096, *Alteromonas* sp. HI0090 and three additional *Alteromonas* isolates (HI0092, HI0107, and HI0109, not shown) were also grown with up to 28.8 μg mL^-1^ of HMWDOM but none had a discernible growth response to the amendment (Supplementary Figure 3).

### Carbon Substrate Utilization Profiles of Isolates

The growth screen and outlier test indicated that isolates closely related to known hydrocarbon degraders, including *Alcanivorax, Oleiphilus, Oleibacter, Thalassolituus*, and *Oleiphilus*, had a strong preference for fatty acids and hydrocarbon-containing compounds, and indicated they can also metabolize low molecular weight (LMW) compounds including acetate and a few carbohydrates (Supplementary Figure 4). Growth of the *Erythrobacter* isolate tested was also stimulated by hydrocarbon-based compounds (Supplementary Figure 4). *Sulfitobacter* sp. HI0054 showed a strong preference for α-amino acids, non-proteinogenic amino acids, and other LMW carbonyl compounds (Supplementary Figure 5). In turn, *P. shioyasakiensis* HI0053 could metabolize simple sugars and polysaccharides, nucleosides, several amino acids, fatty acids, and several other LMW carbonyl compounds (Supplementary Figure 6).

### Representation of Carbohydrate-Degrading Functions in HMWDOM-Enriched Isolates

The number of predicted genes with CAZy domains encoded by HMWDOM-enriched bacterial isolates and model carbohydrate-degrading bacteria is presented in **Table 2**. The analysis focused on identifying CAZy genes with predicted carbohydrate-binding modules (CBM), carbohydrate esterases (CE), glycoside hydrolases (GH), and polysaccharide lyases (PL). In comparison to *Saccharophagus degradans* (Weiner et al., 2008), *Teredinibacter turnerae* (Yang et al., 2009), and *Cellvibrio japonicus* (DeBoy et al., 2008), which encode an unusually large number of carbohydrate-processing enzymes (6–8% of CDS), only 1–2% of the CDSs of the HMWDOM-enriched isolates were predicted to encode CAZy domains. Genes with CE domains accounted for most of the CDSs in these isolates and were comparable to the number of CEs in model carbohydrate-degrading bacteria. The rest of the CAZy families comprised less than 1% of CDSs in the bacterial genomes analyzed.

**Table 2:** The number of predicted carbohydrate-active (CAZy) enzymes in representative bacterial isolates.

Taxon	CBM	CE	GH	PL	CDS
*Saccharophagus degradans^∗^*	136	15	130	33	4008
*Teredinibacter turnerae^∗^*	117	22	101	5	4690
*Cellvibrio japonicus^∗^*	93	19	122	14	3790
*Roseovarius* sp. HI0049	0	28	30	0	5661
*P. shioyasakiensis* HI0053	6	38	53	1	5114
*Oleibacter* sp. HI0075	0	32	14	0	4668
*Thalassolituus* sp. HI0120	2	35	17	2	4424
*Alcanivorax* sps.	0 (0)	33 (8)	13 (3)	1 (1)	3985 (783)
*Erythrobacter* sps.	1 (1)	24 (6)	17 (4)	1 (1)	3206 (602)
*Sulfitobacter* sps.	1 (0)	20 (3)	21 (2)	0 (1)	3947 (244)
*Oleiphilus* sps.	3 (1)	28 (6)	13 (4)	1 (1)	3981 (588)
^∗^Data from Yang et al., 2009					
CDS, coding DNA sequences					

*The number of predicted CAZy genes of bacterial isolates was compared to those of cultured bacteria with known adaptations to degrade complex polysaccharides^∗^. For isolates of the same genus, the means and standard deviations of CAZy genes are indicated. CAZy enzyme families: CBM, carbohydrate-binding modules; CE, carbohydrate esterases; GH, glycoside hydrolases; PL, polysaccharide lyases.*

## Discussion

Our cultivation experiments were motivated by previous work that identified bacterial isolates capable of growth using organic carbon obtained from natural HMWDOM from marine surface waters characteristic of semi-labile DOM (Sosa et al., 2015). We hypothesized that microbial communities inhabiting oligotrophic regions characterized by low primary productivity would harbor bacterial populations adapted to degrade HMWDOM substrates to support their growth.

To this end, we targeted organisms from the lower euphotic zone near the DCM and the upper mesopelagic zone. The DCM at Station ALOHA is situated below the surface mixed layer and coincides roughly with the depth of 1% surface photosynthetically available radiation (Letelier et al., 2004; Laws et al., 2014). At this depth, *Prochlorococcus* makes up approximately 30% of the microbial community and dominates the photosynthetic biomass (Campbell et al., 1997) but primary production rates are three to four times lower than in the surface (Karl and Church, 2014). In turn, in the mesopelagic region where light levels no longer support photosynthesis, the decline of DOC with depth reflects the net biological remineralization of semi-labile DOM (Kaiser and Benner, 2012). Thus, we expected to isolate microorganisms adapted to degrade HMWDOM from both depths, but also expected a lower number of isolates from the DCM since the prevalence of *Prochlorococcus* cells and other photosynthetic organisms at this depth precludes the isolation of other types of organisms by the dilution-to-extinction method.

We found that amending dilution-to-extinction cultures with HMWDOM had a positive effect on the recovery of bacterial isolates. As expected, this effect was greater in mesopelagic samples and was mostly attributed to the enrichment of *Oleiphilus* isolates, 37 of the 58 cultures in these samples (**Figure 1**). In addition, several isolates related to *Sulfitobacter* and *Erythrobacter* were recovered from HMWDOM-amended samples from both depths, suggesting these organisms participate in HMWDOM cycling throughout the lower euphotic zone and mesopelagic region.

### HMWDOM as a Source of Organic Carbon Growth Substrates

Despite the positive effect HMWDOM had on the recovery of dilution-to-extinction cultures, growing these isolates with increasing concentrations of HMWDOM or purified HMWDOM polysaccharides did not stimulate a concomitant response in cell yields (Supplementary Figure 3). *Oleiphilus* and *Sulfitobacter* had the clearest growth response to HMWDOM polysaccharides (**Figure 4**) but the growth difference with respect to unamended media was minimal. Based on previous estimates of bacterial cellular mass (Cermak et al., 2016), a 50% carbon content, and a growth efficiency of 0.5, <1% of the total carbon added as HMWDOM could account for this growth and therefore could represent trace amounts of labile substrates that are co-isolated with HMWDOM polysaccharides (LMW compounds, proteins, etc.) or carbon contamination introduced from sample collection and processing.

This lack of growth on HMWDOM was unexpected and contrasts with the growth response of HMWDOM-enriched OM43 clade methylotrophic bacteria which could reach cell concentrations of up to 6 × 10^6^ cells mL^-1^ with similar HMWDOM amendments (Sosa et al., 2015). Gifford et al. (2016) found that OM43 clade methylotroph strain NB0046 upregulates expression of the methylcitrate cycle, a pathway that characteristically metabolizes propionyl-CoA, when grown in the presence of HMWDOM polysaccharides. This result suggested that compounds associated with HMWDOM polysaccharides were available to these methylotrophs. Whether the fraction of organic carbon in HMWDOM is more limited to our isolates or the specific carbon demands of these isolates and the OM43 clade methylotrophs differ significantly, our results indicate that HMWDOM is a poor source of organic carbon growth substrates to the organisms we investigated.

The overall low representation of genes encoding carbohydrate-degrading functions in our bacterial isolates relative to well-known carbohydrate-degrading bacteria (**Table 2**) and their preference for non-carbohydrate growth substrates (Supplementary Figures 4, 5) is consistent with their minimal response to HMWDOM. Interestingly, however, many of the HMWDOM-enriched isolates had a similar number of predicted CEs as model carbohydrate-degrading bacteria. These enzymes catalyze the removal of ester-based substitutions (de-*O* or de-*N*-acylation) in carbohydrates and thereby facilitate the breakdown of polysaccharides by GHs (Cantarel et al., 2009). This result is consistent with the prevalence of acetyl ester substitutions characteristic of HMWDOM polysaccharides (Aluwihare et al., 1997, 2005) and may indicate that a small fraction of organic carbon in HMWDOM polysaccharides was available to these organisms.

### Bioavailability of Phosphonates in HMWDOM Polysaccharides

The capability to degrade phosphonates is widespread in the marine environment (Villarreal-Chiu et al., 2012). The largest known pools of phosphonates in the marine environment are phosphonates in HMWDOM (Kolowith et al., 2001; Repeta et al., 2016), methylphosphonates derived from marine *Thaumarchaeota* (Metcalf et al., 2012), and phosphonates produced by the cyanobacterium *Trichodesmium* (Dyhrman et al., 2009) and by marine invertebrates (Hilderbrand, 1983). In the NPSG, Martínez et al. (2013) identified bacterial populations related to *Vibrio nigripulchritudo* ATCC27043, the gammaproteobacterium IMCC1989, and the *Rhodobacterales* that express C-P lyase upon phosphate limitation to degrade alkylphosphonates. Furthermore, Repeta et al. (2016) showed that the native microbial community from surface waters in the NPSG and pure cultures of a *P. stutzeri* bacterium encoding C-P lyase can utilize alkylphosphonate esters in HMWDOM polysaccharides to obtain P.

The presence of phosphonate degradation pathways in our HMWDOM-enriched isolates suggested these bacteria might also degrade phosphonate esters in HMWDOM polysaccharides and thus contribute to the production of methane and other hydrocarbons in the ocean. As expected, P-limited cultures of *Sulfitobacter* sp. HI0054 grown with HMWDOM polysaccharides as sole P source produced methane and ethylene (**Table 1**), consistent with the degradation of MPn and 2-HEP esters in HMWDOM (Repeta et al., 2016). The total yield of methane and ethylene (17 nM) in *Sulfitobacter* cultures grown with HMWDOM polysaccharides was 2.2% of the estimated concentration of phosphonates added (780 nM P). These yields are similar to the methane and ethylene produced by P-limited *P. stutzeri* cultures grown with HMWDOM polysaccharides (Repeta et al., 2016).

Previous determinations of the flux of methane to the atmosphere in the NPSG (Holmes et al., 2000) indicate that a minimum methane production rate of 1.7 μmol m^-2^ d^-1^ is necessary to maintain the steady state concentrations observed in the upper 300 m of the water column. Repeta et al. (2016) estimated that a daily turnover of 0.25% of the MPn inventory would be sufficient to support this flux. This translates to an average MPn degradation rate of ∼5.8 × 10^-3^ nmol L^-1^ d^-1^. The MPn in HMWDOM polysaccharides available to *Sulfitobacter* cultures supported a methane production rate of approximately 2 nmol L^-1^ d^-1^, well in excess of this estimate. It is important to note that *Sulfitobacter* growth was stimulated by a concentration of labile organic C (0.2 mM) that may not occur in the environment. Assuming that the MPn degraded per mol of C provided to these cultures (3 × 10^-5^ mol MPn per mol C) is representative of the natural system, <200 nmol C L^-1^ d^-1^ would be sufficient to support the MPn degradation rates necessary to sustain methane supersaturation in the upper ocean. In comparison, heterotrophic production rates in the ocean range from 136.5 nmol C L^-1^ d^-1^ in surface waters (0–200 m depths) to 24.4 nmol C L^-1^ d^-1^ in the mesopelagic region (200–1000 m depths; Arístegui et al., 2009).

Previous studies at Station ALOHA indicate C-P lyase and other phosphonate degradation pathways are prevalent among *Rhodobacterales* populations (Martínez et al., 2010, 2013). The abundance estimates of OTUs representative of the HMWDOM-enriched Roseobacter isolates encoding C-P lyase, including *Sulfitobacter* and *Roseovarius*, were only a small proportion of the microbial community at Station ALOHA, up to 0.24% or 1 out of every ∼400 organisms (Supplementary Table 8). However, C-P lyase appears to be more broadly distributed in members of the Roseobacter clade. Out of 54 Roseobacter genomes searchable in the Roseobase^2^, 38 encode proteins highly similar to the C-P lyase pathway catalytic proteins of *Sulfitobacter* sp. HI0054 (PhnI, 77–95% amino acid identity; PhnJ, 83–93%; PhnK, 85–99%; and PhnL, 71–97%). Assuming that members of this clade generally encode C-P lyase, they may account for most of the C-P lyase identified at Station ALOHA (1 copy in every 300–400 average genomes). Presumably, a much higher diversity of bacteria known to encode C-P lyase (Villarreal-Chiu et al., 2012), including our *Oleiphilus* isolates and more abundant clades such as SAR11 (Carini et al., 2014) that were not captured by our culturing experiments, contribute to this signal. Our findings nevertheless further associate members of the Roseobacter clade with phosphonate cycling and with the aerobic production of methane in the ocean.

Given that *Sulfitobacter* cultures grown with HMWDOM polysaccharides reached similar cell densities as cultures amended with alkylphosphonates (**Table 1**), it is possible that *Sulfitobacter* utilized additional forms of organic P available in HMWDOM polysaccharides. Phosphonates comprise only 20% of the total P in HMWDOM polysaccharides. Assuming the average methane, ethylene, and ethane yields in MPn, 2-HEP, and EPn treatments, respectively (∼223 nM), approximates the P demand of these cultures, about 200 nM P was obtained from phosphate esters and diesters or other P compounds in HMWDOM polysaccharides. This assumes also that the cellular C:P ratio of these cultures did not change.

Owing to the phosphate-dependent regulation of C-P lyase by the Pho regulon (Chen et al., 1990; Metcalf and Wanner, 1993a), we postulate that the hydrolysis of the ester bond between phosphonates and HMWDOM polysaccharides is mediated by enzymes similar to alkaline phosphatase (APase). The *P. stutzeri* strain HI00D01 used by Repeta et al. (2016) encodes an APase of the PhoD family (KZX59646.1). *Sulfitobacter* sp. HI0054 also encodes a PhoD-like APase (KZY51209.1) and a related enzyme (KZY52437.1) annotated as a phosphonate monoester hydrolase (PEH). This PEH was present in the genomes of 7 of the 8 *Sulfitobacter* isolates with annotated genomes, including all those containing C-P lyase. PEH has been characterized in *Burkholderia caryophilli* PG2982 (Dotson et al., 1996) and in *Rhizobium leguminosarum* (Jonas et al., 2008) as having high substrate specificity to phosphonate monoesters and phosphodiesters, the two major P functional groups in HMWDOM. The PEH of *Sulfitobacter* sp. HI0054 shared 31 and 34% amino acid sequence similarity with the PEH of *R. leguminosarum* and *B. caryophilli*, respectively. The isolation of these phosphonate degraders will allow us to explore the role of marine APases in the degradation of HMWDOM phosphonates and other HMWDOP substrates.

### Links between Methane, Phosphonate Degradation, and Polysaccharide Cycling in the NPSG

In the NPSG, methane is typically supersaturated with respect to equilibrium concentrations with the atmosphere in the upper 300 m (**Figure 3B**; Tilbrook and Karl, 1995; Holmes et al., 2000). Luo et al. (2011) reported that C-P lyase is mostly present in surface waters (<70 m) in the NPSG and is unlikely to occur below depths >130 m. However, our metagenomic analysis at Station ALOHA identified C-P lyase pathway genes in all samples between the surface and 1000 m depths (**Figure 3A**). We also detected the highest abundance and variability of C-P lyase in the lower euphotic zone and upper mesopelagic region (**Figure 3A**). Our observations were in agreement with the abundance estimates of *phnI* and *phnJ* reported by Martínez et al. (2010) of <1% of organisms in samples between the surface and 500 m at Station ALOHA. It is possible that the detection of C-P lyase genes by Luo et al. (2011) was limited by the amount of metagenomic sequence data available (∼64 Mbps compared to 456 Gbp in the ALOHA gene catalog).

While the profile of dissolved methane with depth merely reflects steady state concentrations, the presence of C-P lyase in mesopelagic isolates and the increase in C-P lyase pathway genes at these depths are a strong indication that phosphonate cycling is important up to at least 200–300 m. The proportions of phosphonates and phosphate esters in HMWDOP remain relatively stable throughout surface, mesopelagic, and bathypelagic waters in the Pacific Ocean (Clark et al., 1998), indicating these two P pools cycle at similar rates. These observations are consistent with the utilization of phosphonates in HMWDOM polysaccharides and the presence of C-P lyase throughout the water column, suggesting that MPn is not only an important source of methane in the upper ocean but may be a source of methane to the deep sea as well.

The preferential utilization of DOP in phosphate-replete environments like the mesopelagic zone suggests that organisms degrade organic P compounds for reasons other than to acquire phosphate (Karl and Björkman, 2015). Increased APase activity in the mesopelagic waters relative to surface waters, for example, has been associated with the bacterial degradation of DOP to obtain not only P but also C for energy and growth (Hoppe and Ullrich, 1999). One hypothesis that may explain the occurrence of C-P lyase in phosphate-replete depths in the ocean is that phosphonate substitutions in HMWDOM polysaccharides make these inaccessible to enzymes that break down carbohydrates. This protection would be a natural function of phosphonates in cells. Selection for phosphonate-degrading bacteria and for C-P lyase at these depths may therefore be driven by the need of the microbial community to access the chemical energy stored in phosphonate-bounded carbohydrates. Additionally, some phosphonate degraders may be localized on sinking particles as suggested by del Valle and Karl (2014) and would therefore be transported from the surface to the deep sea. Deeper in the mesopelagic region, the decline of methane concentrations from 300 to 1000 m depths (**Figure 3B**) and the enrichment of ^13^C-methane with depth (Holmes et al., 2000) suggests that methane-oxidizing organisms may benefit from the degradation of MPn stored in HMWDOM polysaccharides. Thus, C-P lyase would be valuable for the community at these depths as well.

## Conclusion

This study investigated the potential of HMWDOM to serve as a source of carbon and phosphorus to marine bacteria inhabiting the open ocean environment. HMWDOM appears to be a poor source of carbon for monocultures of the bacteria we isolated. Organic phosphorus compounds in HMWDOM, however, like the alkylphosphonates metabolized by *Sulfitobacter* cultures, do seem more readily available and likely represent an important source of P to marine bacteria. Our findings, combined with previous observations, add support to the role of MPn stored in HMWDOM as a key methanogenic substrate in the aerobic ocean. The lack of growth response we observed in bacterial cultures supplemented with HMWDOM possibly reflects the semi-labile nature of this material and the complex microbial processing and inter-species interactions that may be required to mediate its turnover. It is also possible that some of the more abundant bacterial clades in the oligotrophic ocean contribute significantly to the degradation of semi-labile DOM, but our culturing conditions could not support these. Due to the high representation of phosphonate-degrading bacteria in our samples, we postulate that the degradation of phosphonate ester substitutions in HMWDOM polysaccharides is a key biochemical step in the turnover of semi-labile DOM. The metabolic properties of the isolates obtained in this study will be explored further to investigate the degradation pathways of semi-labile DOM and marine phosphonates.

## Author Contributions

OS, DR, DK, and ED designed research. OS performed research. OS and SF performed gas analysis. JB and DM provided metagenomic analyses. DR isolated and processed the HMWDOM. OS, DR, DK, and ED wrote the paper with contributions from all authors.

## Conflict of Interest Statement

The authors declare that the research was conducted in the absence of any commercial or financial relationships that could be construed as a potential conflict of interest.

## Footnotes

1 http://hahana.soest.hawaii.edu/hoeboe/hoeboe.html

2 http://www.roseobase.org
