# Supplementary material for: Isolation and Characterization of Bacteria That Degrade Phosphonates in Marine Dissolved Organic Matter
Source: Front Microbiol. 2017 Sep 26;8:1786. doi: 10.3389/fmicb.2017.01786 (PMC5649143; doi:10.3389/fmicb.2017.01786)
Supplement: Supplementary file 1 [file Data_Sheet_1.PDF]

Supplementary Material

Isolation and Characterization of Bacteria that Degrade Phosphonates in Marine Dissolved Organic Matter

Oscar A. Sosa*, Daniel J. Repeta, Sara Ferrón, Jessica A. Bryant, Daniel R. Mende, David. M. Karl, and Edward F. DeLong*

***Correspondence:** Edward DeLong: edelong@hawaii.edu, Oscar Sosa: ososa@hawaii.edu

Supplementary Table 1. The three C-P lyase gene clusters characterized by Martínez et al. (2013) in microcosm experiments amended with methylphosphonate. The amino acid sequences of these genes were used as queries to find closely related sequences in the genomes of bacteria isolated from dilution-to-extinction cultures.

B3TF_MPn1 gammaproteobacterium IMCC1989		B3TF_MPn2 *Rhodobacterales*		B3TF_MPn8 *Vibrio nigripulchritudo* ATCC27043	
**C-P lyase protein**	**GI accession**	**C-P lyase protein**	**GI accession**	**C-P lyase protein**	**GI accession**
PT	AHG53029.1	PhnC	AHG53005.1	PhnC	AHG52968.1
PhnN	AHG53030.1	PhnD	AHG53006.1	PhnD	AHG52969.1
PhnM	AHG53031.1	PhnE1	AHG53007.1	PhnE	AHG52970.1
PhnL	AHG53032.1	PhnE2	AHG53008.1	PhnF	AHG52971.1
PhnK	AHG53033.1	PT	AHG53009.1	PhnG	AHG52972.1
PhnJ	AHG53034.1	PhnM1	AHG53010.1	PhnH	AHG52973.1
PhnI	AHG53035.1	PhnF	AHG53011.1	PhnI	AHG52974.1
PhnH	AHG53036.1	PhnG	AHG53012.1	PhnJ	AHG52975.1
PhnG	AHG53037.1	PhnH	AHG53013.1	PhnK	AHG52976.1
PhnE	AHG53038.1	PhnI	AHG53014.1	PhnL	AHG52977.1
PhnD	AHG53039.1	PhnJ	AHG53016.1	PhnM	AHG52978.1
PhnC	AHG53040.1	PhnK	AHG53017.1	PhnN	AHG52979.1
PAT	AHG53041.1	PhnL	AHG53018.1	PhnP	AHG53043.1
PhnF	AHG53042.1	PhnN	AHG53019.1		
		RcsF	AHG53020.1		
		PhnM2	AHG53021.1		

Abbreviations: GI, GenBank identifier; PT, putative transferase; PAT, putative acyl-CoA N-acetyltransferase

Supplementary Table 2. Vitamin additions to *Sulfitobacter sp*. HI0054 growth medium.

Vitamin	Final concentration (nM)
Pyrodxine-HCl	243
Clacium pantothenate	420
PABA-paraaminobenzoic acid	36
Folic acid	2
Vitamin B12	0.4
Biotin	2
Thiamine	297

Supplementary Table 3. List of treatments used in the dilution-to-extinction experiments as well as total organic carbon (TOC) added with HMWDOM amendments.

Treatment	Description	HMWDOM added (mg L^{-1})	TOC added (µM)	Final TOC (µM)
I	Unamended controls	-	-	67.4
II	Ultrafiltered HMWDOM	7.2	162.3	229.7
III	Ultrafiltered HMWDOM	18	404.8	472.2
IV	HMWDOM polysaccharides	7.2	251.6	319.0

Supplementary Table 4. Distribution of cell yields and number of cultures recovered after screening dilution-to-extinction cultures by flow cytometry. Treatments I-IV correspond to those described in Table 1. Wells that tested positive for growth (>$1x10^4$ cells ml^{-1}) in each treatment are indicated in the highlighted area.

Treatment	Sterile culturing media				DCM (95 m)				Mesopelagic (250 m)			
	I	II	III	IV	I	II	III	IV	I	II	III	IV
**Cell count (per mL)**												
<10^3	141	143	139	144	694	710	705	704	704	683	667	662
10^3 to $5x10^3$	3	1	5	0	19	4	4	6	11	23	15	12
$5x10^3$ to 10^4	0	0	0	0	1	0	2	0	0	2	12	6
10^4 to $5x10^4$	0	0	0	0	2	4	4	4	3	6	6	13
$5x10^4$ to 10^5	0	0	0	0	1	0	2	1	2	2	7	9
10^5 to $5x10^5$	0	0	0	0	2	1	2	4	0	3	10	12
$5x10^5$ to 10^6	0	0	0	0	0	0	0	1	0	0	2	2
10^6 to $5x10^6$	0	0	0	0	1	1	1	0	0	1	1	4
Total wells	144	144	144	144	720	720	720	720	720	720	720	720
**Percent Recovery**												
>10^4	0.00	0.00	0.00	0.00	0.83	0.83	1.25	1.39	0.69	1.67	3.61	5.56

Supplementary Table 5. Genome assembly summary of 55 representative bacterial isolates. Annotated genome assemblies were deposited under NCBI BioProject PRJNA305749 with GenBank accessions LWEI00000000-LWGK00000000.

Isolate ID	Organism name	GenBank accession	Reads assembled	%GC	Coverage[a]	Total contigs	N50	Total consensus	Quality[b]
HI0003	*Alcanivorax sp.* HI0003	LWEI00000000	552,606	59	27	378	25,724	3,635,447	81
HI0007	*Alcanivorax sp.* HI0007	LWEJ00000000	716,839	59	35	286	46,304	3,620,498	81
HI0009	*Oleiphilus sp.* HI0009	LWEK00000000	723,138	44	30	2,650	16,347	6,316,839	55
HI0011	*Alcanivorax sp.* HI0011	LWEL00000000	627,875	59	26	2,482	21,670	5,632,014	58
HI0013	*Alcanivorax sp.* HI0013	LWEM00000000	850,375	59	36	3,199	18,983	6,607,668	60
HI0019	*Erythrobacter sp.* HI0019	LWEN00000000	320,380	64	18	880	8,707	3,344,147	81
HI0020	*Erythrobacter sp.* HI0020	LWEO00000000	586,146	63	32	275	41,642	3,189,298	81
HI0021	*Sulfitobacter sp.* HI0021	LWEP00000000	631,044	61	24	717	18,129	4,462,051	81
HI0023	*Sulfitobacter sp.* HI0023	LWEQ00000000	596,480	63	26	652	18,405	4,303,594	81
HI0027	*Sulfitobacter sp.* HI0027	LWER00000000	1,124,339	61	45	458	46,668	4,441,089	81
HI0028	*Erythrobacter sp.* HI0028	LWES00000000	324,735	64	19	610	12,790	3,308,918	44
HI0033	*Alcanivorax sp.* HI0033	LWET00000000	488,095	59	25	509	20,669	3,659,598	81
HI0035	*Alcanivorax sp.* HI0035	LWEU00000000	822,176	59	35	3,128	17,383	6,690,527	59
HI0037	*Erythrobacter sp.* HI0037	LWEV00000000	774,140	63	37	2,993	21,899	5,769,061	62
HI0038	*Erythrobacter sp.* HI0038	LWEW00000000	475,039	63	22	2,004	14,945	4,656,539	58
HI0040	*Sulfitobacter sp.* HI0040	LWEX00000000	646,904	63	27	611	20,422	4,181,428	81
HI0043	*Oleiphilus sp.* HI0043	LWEY00000000	707,561	44	26	1,975	25,101	5,472,755	54
HI0044	*Alcanivorax sp.* HI0044	LWEZ00000000	488,420	58	19	1,990	10,187	5,153,888	60
HI0049	*Roseovarius sp.* HI0049	LWFA00000000	567,627	64	16	3,470	4,966	7,096,493	66
HI0050	*Oleiphilus sp.* HI0050	LWFB00000000	344,906	44	14	2,036	5,000	4,819,895	71
HI0053	*P. shioyasakiensis* HI0053	LWFC00000000	695,383	42	19	2,964	5,342	6,718,721	70
HI0054	*Sulfitobacter sp.* HI0054	LWFD00000000	731,891	62	31	555	23,429	4,174,729	81
HI0061	*Oleiphilus sp.* HI0061	LWFE00000000	434,002	44	18	1,885	8,507	5,095,272	60
HI0063	*Erythrobacter sp.* HI0063	LWFF00000000	630,550	63	33	356	41,846	3,258,146	81
HI0065	*Oleiphilus sp.* HI0065	LWFG00000000	818,810	48	35	2,693	28,355	6,398,614	56
HI0066	*Oleiphilus sp.* HI0066	LWFH00000000	560,893	44	25	1,887	22,586	5,044,867	54
HI0067	*Oleiphilus sp.* HI0067	LWFI00000000	503,329	44	22	1,752	20,427	4,840,075	55
HI0068	*Oleiphilus sp.* HI0068	LWFJ00000000	365,219	44	14	2,001	5,172	4,842,151	71
HI0069	*Oleiphilus sp.* HI0069	LWFK00000000	590,352	44	22	2,238	14,127	5,901,752	53
HI0071	*Oleiphilus sp.* HI0071	LWFL00000000	601,234	48	26	2,289	24,676	5,698,317	53
HI0072	*Oleiphilus sp.* HI0072	LWFM00000000	337,845	44	14	2,192	4,687	4,921,543	71
HI0073	*Oleiphilus sp.* HI0073	LWFN00000000	784,137	48	34	2,628	56,040	6,103,244	55
HI0074	*Erythrobacter sp.* HI0074	LWFO00000000	379,698	64	22	583	12,646	3,148,101	81
HI0075	*Oleibacter sp.* HI0075	LWFP00000000	535,677	51	22	2,573	12,664	6,118,559	56
HI0076	*Sulfitobacter sp.* HI0076	LWFQ00000000	574,934	61	23	875	13,538	4,370,296	81
HI0077	*Erythrobacter sp.* HI0077	LWFR00000000	365,940	64	21	592	13,723	3,161,113	81
HI0078	*Oleiphilus sp.* HI0078	LWFS00000000	303,674	44	12	2,530	2,905	4,431,832	78
HI0079	*Oleiphilus sp.* HI0079	LWFT00000000	426,827	47	19	1,448	13,007	4,407,017	58
HI0080	*Oleiphilus sp.* HI0080	LWFU00000000	377,861	47	16	1,249	8,128	3,938,001	72
HI0081	*Oleiphilus sp.* HI0081	LWFV00000000	298,111	44	12	2,463	3,030	4,402,732	78
HI0082	*Sulfitobacter sp*. HI0082	LWFW00000000	616,668	61	21	2,310	9,483	5,443,144	62
HI0083	*Alcanivorax sp.* HI0083	LWFX00000000	345,236	58	14	1,795	5,595	4,446,909	73
HI0085	*Oleiphilus sp.* HI0085	LWFY00000000	253,579	44	11	3,230	2,080	4,482,542	78
HI0086	*Oleiphilus sp.* HI0086	LWFZ00000000	506,633	44	20	1,534	10,771	4,753,747	63
HI0117	*Oleiphilus sp.* HI0117	LWGA00000000	546,950	44	19	1,521	7,176	4,620,570	75
HI0118	*Oleiphilus sp.* HI0118	LWGB00000000	1,204,101	48	49	2,826	15,926	6,560,891	55
HI0120	*Thalassolituus sp.* HI0120	LWGC00000000	766,145	49	27	2,071	35,705	5,674,715	55
HI0122	*Oleiphilus sp.* HI0122	LWGD00000000	743,601	48	32	2,490	37,188	6,039,240	55
HI0123	*Oleiphilus sp.* HI0123	LWGE00000000	545,524	44	17	1,909	5,022	4,468,126	79
HI0125	*Oleiphilus sp.* HI0125	LWGF00000000	605,193	44	25	1,531	19,709	4,553,828	55
HI0128	*Oleiphilus sp.* HI0128	LWGG00000000	568,083	44	20	1,636	11,631	4,885,910	59
HI0129	*Sulfitobacter sp.* HI0129	LWGH00000000	395,966	63	17	1,145	8,517	4,210,493	81
HI0130	*Oleiphilus sp.* HI0130	LWGI00000000	433,776	48	18	2,064	10,326	5,194,401	57
HI0132	*Oleiphilus sp.* HI0132	LWGJ00000000	501,301	44	20	1,091	9,473	5,170,237	61
HI0133	*Oleiphilus sp.* HI0133	LWGK00000000	869,569	48	36	2,393	47,727	5,728,633	55

[a]Average total coverage assessment calculated from contigs >= 5000 with uniform coverage
[b]Average concensus quality

Supplementary Table 6. Genome assembly summary and gene composition of 55 representative bacterial isolates. The number of protein-coding genes, rRNAs, tRNAs, and partial rRNA genes are indicated. Annotated genome assemblies were deposited under NCBI BioProject PRJNA305749 with GenBank accessions LWEI00000000-LWGK00000000.

Isolate ID	Organism name	GenBank accession	Total genes	CDS[a]	5S[b]	16S[b]	23S[b]	tRNAs	Partial 16S[c]	%ID[d]
HI0003	*Alcanivorax sp.* HI0003	LWEI00000000	3,523	3,464	3	1	1	45	3	100
HI0007	*Alcanivorax sp.* HI0007	LWEJ00000000	3,485	3,196	2	1	1	46	4	99-100
HI0009	*Oleiphilus sp.* HI0009	LWEK00000000	7,684	5,156	6	2	1	108	2	100
HI0011	*Alcanivorax sp.* HI0011	LWEL00000000	6,688	4,358	4	1	-	75	6	100
HI0013	*Alcanivorax sp.* HI0013	LWEM00000000	7,978	5,037	4	1	1	84	5	100
HI0019	*Erythrobacter sp.* HI0019	LWEN00000000	3,719	2,916	1	1	-	49	1	100
HI0020	*Erythrobacter sp.* HI0020	LWEO00000000	3,265	2,930	2	1	1	46	1	100
HI0021	*Sulfitobacter sp.* HI0021	LWEP00000000	4,750	3,982	4	1	1	47	6	99-100
HI0023	*Sulfitobacter sp.* HI0023	LWEQ00000000	4,560	3,909	1	1	-	44	0	-
HI0027	*Sulfitobacter sp.* HI0027	LWER00000000	4,587	4,036	4	1	1	46	5	99-100
HI0028	*Erythrobacter sp.* HI0028	LWES00000000	3,546	2,939	1	1	1	49	0	-
HI0033	*Alcanivorax sp.* HI0033	LWET00000000	3,620	3,184	3	1	-	45	3	99-100
HI0035	*Alcanivorax sp.* HI0035	LWEU00000000	8,050	5,101	3	2	1	89	4	100
HI0037	*Erythrobacter sp.* HI0037	LWEV00000000	7,365	4,492	3	2	1	89	0	100
HI0038	*Erythrobacter sp.* HI0038	LWEW00000000	5,730	3,759	2	1	1	75	3	100
HI0040	*Sulfitobacter sp.* HI0040	LWEX00000000	4,419	3,813	1	1		44	2	100
HI0043	*Oleiphilus sp.* HI0043	LWEY00000000	6,082	4,252	6	2	1	59	0	100
HI0044	*Alcanivorax sp.* HI0044	LWEZ00000000	5,863	4,061	4	2	1	63	2	100
HI0049	*Roseovarius sp.* HI0049	LWFA00000000	8,752	5,661	1	-	-	59	6	92-100
HI0050	*Oleiphilus sp.* HI0050	LWFB00000000	5,476	3,654	5	1	1	49	6	100
HI0053	*P. shioyasakiensis* HI0053	LWFC00000000	7,526	5,114	15	2	2	140	14	99-100
HI0054	*Sulfitobacter sp.* HI0054	LWFD00000000	4,363	3,790	4	1	1	43	3	99-100
HI0061	*Oleiphilus sp.* HI0061	LWFE00000000	5,727	3,986	4	1	1	57	4	99-100
HI0063	*Erythrobacter sp.* HI0063	LWFF00000000	3,299	2,924	1	-	1	48	4	100
HI0065	*Oleiphilus sp.* HI0065	LWFG00000000	7,425	4,935	6	2	1	77	3	100
HI0066	*Oleiphilus sp.* HI0066	LWFH00000000	5,809	4,024	6	1	1	59	2	100
HI0067	*Oleiphilus sp.* HI0067	LWFI00000000	5,576	3,903	5	2	1	64	0	100
HI0068	*Oleiphilus sp.* HI0068	LWFJ00000000	5,548	3,709	5	1	2	53	6	99-100
HI0069	*Oleiphilus sp.* HI0069	LWFK00000000	6,607	4,573	5	2	2	66	1	99-100
HI0071	*Oleiphilus sp.* HI0071	LWFL00000000	6,536	4,361	6	1	2	72	4	100
HI0072	*Oleiphilus sp.* HI0072	LWFM00000000	5,710	3,712	4	1	-	52	5	96-100
HI0073	*Oleiphilus sp.* HI0073	LWFN00000000	7,136	4,664	8	2	2	75	4	100
HI0074	*Erythrobacter sp.* HI0074	LWFO00000000	3,382	2,827	2	1	-	48	0	-
HI0075	*Oleibacter sp.* HI0075	LWFP00000000	7,198	4,668	5	1	2	89	7	99-100
HI0076	*Sulfitobacter sp.* HI0076	LWFQ00000000	4,754	3,879	3	1	1	46	6	99-100
HI0077	*Erythrobacter sp.* HI0077	LWFR00000000	3,385	2,859	2	1	1	48	1	100
HI0078	*Oleiphilus sp.* HI0078	LWFS00000000	5,461	3,209	6	1	1	40	4	99-100
HI0079	*Oleiphilus sp.* HI0079	LWFT00000000	4,941	3,504	5	2	2	51	1	100
HI0080	*Oleiphilus sp.* HI0080	LWFU00000000	4,329	3,158	4	1	1	54	5	99-100
HI0081	*Oleiphilus sp.* HI0081	LWFV00000000	5,397	3,187	5	1	1	51	4	99-100
HI0082	*Sulfitobacter sp*. HI0082	LWFW00000000	6,566	4,483	5	2	1	69	5	100
HI0083	*Alcanivorax sp.* HI0083	LWFX00000000	5,170	3,476	3	1	1	55	6	99-100
HI0085	*Oleiphilus sp.* HI0085	LWFY00000000	5,916	3,016	5	1	1	51	4	99-100
HI0086	*Oleiphilus sp.* HI0086	LWFZ00000000	5,145	3,741	6	2	2	54	2	99-100
HI0117	*Oleiphilus sp.* HI0117	LWGA00000000	4,989	3,665	5	1	1	54	2	100
HI0118	*Oleiphilus sp.* HI0118	LWGB00000000	7,665	5,064	7	2	2	87	3	100
HI0120	*Thalassolituus sp.* HI0120	LWGC00000000	6,410	4,424	7	2	1	81	10	99-100
HI0122	*Oleiphilus sp.* HI0122	LWGD00000000	6,965	4,622	7	2	1	76	4	100
HI0123	*Oleiphilus sp.* HI0123	LWGE00000000	5,056	3,413	5	1	1	49	3	100
HI0125	*Oleiphilus sp.* HI0125	LWGF00000000	5,107	3,684	4	2	1	50	2	100
HI0128	*Oleiphilus sp.* HI0128	LWGG00000000	5,320	3,811	4	1	1	49	3	100
HI0129	*Sulfitobacter sp.* HI0129	LWGH00000000	4,753	3,681	1	-	-	42	4	100
HI0130	*Oleiphilus sp.* HI0130	LWGI00000000	6,013	4,079	5	2	2	66	2	99-100
HI0132	*Oleiphilus sp.* HI0132	LWGJ00000000	5,733	4,071	4	1	1	55	4	99-100
HI0133	*Oleiphilus sp.* HI0133	LWGK00000000	6,642	4,339	6	2	1	79	3	100

[a]Protein coding DNA sequences
[b]Complete rRNA genes
[c]Number of partial 16S rRNA gene sequences identified
[d]Percent identity between partial 16S rRNA sequences and the full-length sequences identified

Supplementary Table 7. SSU rRNA gene phylogeny of dilution-to-extinction cultures and best matching described taxa. This table summarizes the dilution-to-extinction sample and treatment from which cultures were obtained as well as the number of cultures with the same SSU rRNA gene sequence similarity to a SILVA reference sequence.

Isolate ID[a]	Best matching described taxa via SSU rRNA[b]	%ID[c]	Length[d]	No. cultures[e]	Treatment[f]
**Deep chlorophyll maximum**					
9	*Oleiphilus*;uncultured bacterium AD12-E7	98.0	1514	1	I
21	*Oceanibulbus indolifex* HEL-45	99.5	1444	1	III
27	*Oceanibulbus indolifex* HEL-45	99.5	1219	1	III
19, 28	*Erythrobacter*;uncultured bacterium AD84-H10	99.9	1468	2	III
23, 40	*Oceanibulbus*; marine bacterium SCRIPPS_101	98.1	1437	2	III & IV
20, 37, 38	*Erythrobacter*; uncultured bacterium (EF659424.1.1483)	99.9	1453	3	III & IV
3, 7, 11, 13, 33, 35	*Alcanivorax sp.* Abu-1	99.9	1507	6	I, II, & IV
**Mesopelagic**					
43[g]	*Oleiphilus*; uncultured gamma proteobacterium (JQ579692.1.1494)	98.2	1494	16	I
49	*Roseovarius*; uncultured alpha proteobacterium (HQ727624.1.1431)	99.8	1431	1	II
53	*Pseudoalteromonas*; uncultured bacterium (KF906589.1.1533)	99.5	1509	1	II
54	*Sulfitobacter sp.* CC-AMSY-48	99.9	1429	1	II
63	*Erythrobacter*; uncultured bacterium HF0500_24B12	100.0	1469	1	II
65[h]	*Oleiphilus*; uncultured bacterium AD12-E7	99.3	1514	15	III
75	*Oleibacter*; uncultured bacterium (AF382127.1.1491)	99.7	1491	1	III
76	*Oceanibulbus indolifex* HEL-45	99.5	1300	1	III
82	*Oceanibulbus indolifex* HEL-45	99.4	1444	1	III
90	*Alteromonas sp.* KOPRI 11568	99.8	1276	1	IV
92	*Alteromonas macleodii* str. 'Black Sea 11'	98.5	819	1	IV
96	*Alcanivorax sp.* Mho1	99.9	1435	1	IV
97	*Oceanibulbus indolifex* HEL-45	99.5	1219	1	IV
107	uncultured *Psychrobacter sp.* (JX530017.1.1434)	82.6	1113	1	IV
109	*Alteromonas macleodii* str. 'Black Sea 11'	100.0	1187	1	IV
113	*Oleibacter*; uncultured bacterium (AF382127.1.1491)	99.8	1491	1	IV
120	*Thalassolituus*; uncultured gamma proteobacterium (FJ403082.1.1500)	97.9	1500	1	IV
66, 67	*Oleiphilus*;uncultured bacterium AD12-E7	98.0	1514	2	III
74, 77	*Erythrobacter*; uncultured bacterium AD84-H10	99.9	1468	2	IIII
44, 83	*Alcanivorax*; unidentified (E31375.1.1516)	99.5	1516	2	I & III
69, 85	*Oleiphilus*; uncultured gamma proteobacterium (JQ579692.1.1494)	98.1	1494	2	III
94, 125	*Oleiphilus*; uncultured bacterium AD12-E7	97.9	1514	2	IV
112, 129	*Oceanibulbus*; marine bacterium SCRIPPS_101	98.1	1437	2	IV

[a]Culture identification number (HI0###).

[b]Top hit of a LAST sequence similarity search against the SILVA 119 SSU database.

[c]Percent matching identities to SILVA reference sequence

[d]Alignment length

[e]Number of cultures with equal SSU rRNA gene percent matching identities

[f]HMWDOM amendments to dilution-to-extinction samples: treatment I: unamended control samples; treatment II: 7.2 mg L^{-1}; treatment III 18 mg L^{-1}; treatment IV: 7.2 mg L^{-1} (purified HMWDOM polysaccharides)

[g]Isolate IDs (HI0###) matching HI0043: 050, 061, 068, 072, 078, 081, 086, 098, 100, 103, 111, 117, 123, 128, and 132

[h]Isolate IDs (HI0###) mathcing HI0065: 071, 073, 079, 080, 087, 088, 104, 105, 108, 114, 118, 122, 130, 133

Supplementary Table 8. The abundance of OTUs representative of bacteria isolated at Station ALOHA. Abundance estimates were normalized by the average genome size in the metagenome and are expressed as the percent (mean and standard deviation) of the total community. The number of isolates mapped to each OTU is indicated in parenthesis.

	**25 m**	**45 m**	**75 m**	**125 m**	**200 m**	**500 m**	**770 m**	**1000 m**
Alcanivorax sp. HI0003 (1)	0.099(0.047)	0.084(0.044)	0.103(0.035)	0.074(0.037)	0.073(0.041)	0.059(0.024)	0.046(0.015)	0.066(0.046)
Alcanivorax sp. HI0011 (4)	0.003(0.004)	0.009(0.018)	0.003(0.004)	0.005(0.005)	0.009(0.007)	0.008(0.006)	0.01(0.009)	0.011(0.014)
Alteromonas sp. HI0090 (3)	0.001(0.003)	0.001(0.002)	0.001(0.002)	0.041(0.091)	0.064(0.129)	0.85(1.718)	0.225(0.238)	0.468(0.67)
Erythrobacter sp. HI0019 (6)	0.184(0.082)	0.208(0.095)	0.191(0.089)	0.151(0.051)	0.132(0.049)	0.141(0.057)	0.162(0.078)	0.322(0.534)
Erythrobacter sp. HI0038 (2)	0.191(0.088)	0.179(0.113)	0.217(0.116)	0.251(0.124)	0.363(0.123)	0.269(0.087)	0.387(0.193)	0.555(0.848)
Sulfitobacter sp. HI0021 (7)*	0.003(0.003)	0.007(0.006)	0.004(0.005)	0.007(0.004)	0.017(0.014)	0.014(0.008)	0.021(0.026)	0.025(0.025)
Sulfitobacter sp. HI0076 (3)*	0.226(0.095)	0.241(0.104)	0.23(0.081)	0.215(0.073)	0.241(0.079)	0.201(0.057)	0.241(0.089)	0.221(0.122)
Oleibacter sp. HI0075 (1)	0.004(0.004)	0(0.001)	0.004(0.005)	0.004(0.005)	0.003(0.006)	0.005(0.006)	0.003(0.003)	0.005(0.005)
Oleibacter sp. HI0113 (1)	0.006(0.007)	0.007(0.007)	0.005(0.006)	0.006(0.006)	0.011(0.012)	0.034(0.066)	0.027(0.033)	0.029(0.032)
Oleiphilus sp. HI0009 (4)*	0.006(0.006)	0(0)	0.004(0.005)	0.002(0.002)	0.007(0.009)	0.009(0.009)	0.008(0.009)	0.017(0.015)
Oleiphilus sp. HI0043 (6)	0.005(0.007)	0.003(0.003)	0.004(0.004)	0.006(0.005)	0.017(0.015)	0.086(0.189)	0.036(0.083)	0.185(0.318)
Oleiphilus sp. HI0050 (12)	0.003(0.004)	0.006(0.007)	0.004(0.005)	0.003(0.003)	0.008(0.01)	0.004(0.004)	0.003(0.004)	0.005(0.004)
Oleiphilus sp. HI0065 (16)*	0.015(0.01)	0.014(0.01)	0.016(0.009)	0.038(0.032)	0.092(0.037)	0.092(0.036)	0.087(0.043)	0.127(0.093)
P. shioyasakiensis HI0053 (1)	0.018(0.008)	0.007(0.003)	0.016(0.012)	0.026(0.023)	0.076(0.082)	0.347(0.535)	0.189(0.247)	0.42(0.431)
Psychrobacter sp. HI0107 (1)	0.002(0.003)	0.003(0.003)	0.001(0.002)	0.017(0.024)	0.007(0.018)	0.023(0.038)	0.016(0.038)	0.026(0.029)
Roseovarius sp. HI0049 (1)*	0.002(0.004)	0.001(0.002)	0.003(0.006)	0.002(0.005)	0.002(0.003)	0.001(0.002)	0.004(0.005)	0.004(0.004)
Thalassolituus sp. HI0120 (1)	0.011(0.007)	0.007(0.005)	0.009(0.007)	0.012(0.01)	0.014(0.02)	0.035(0.062)	0.017(0.018)	0.028(0.02)

*OTUs that inlcude strains with C-P lyase operons

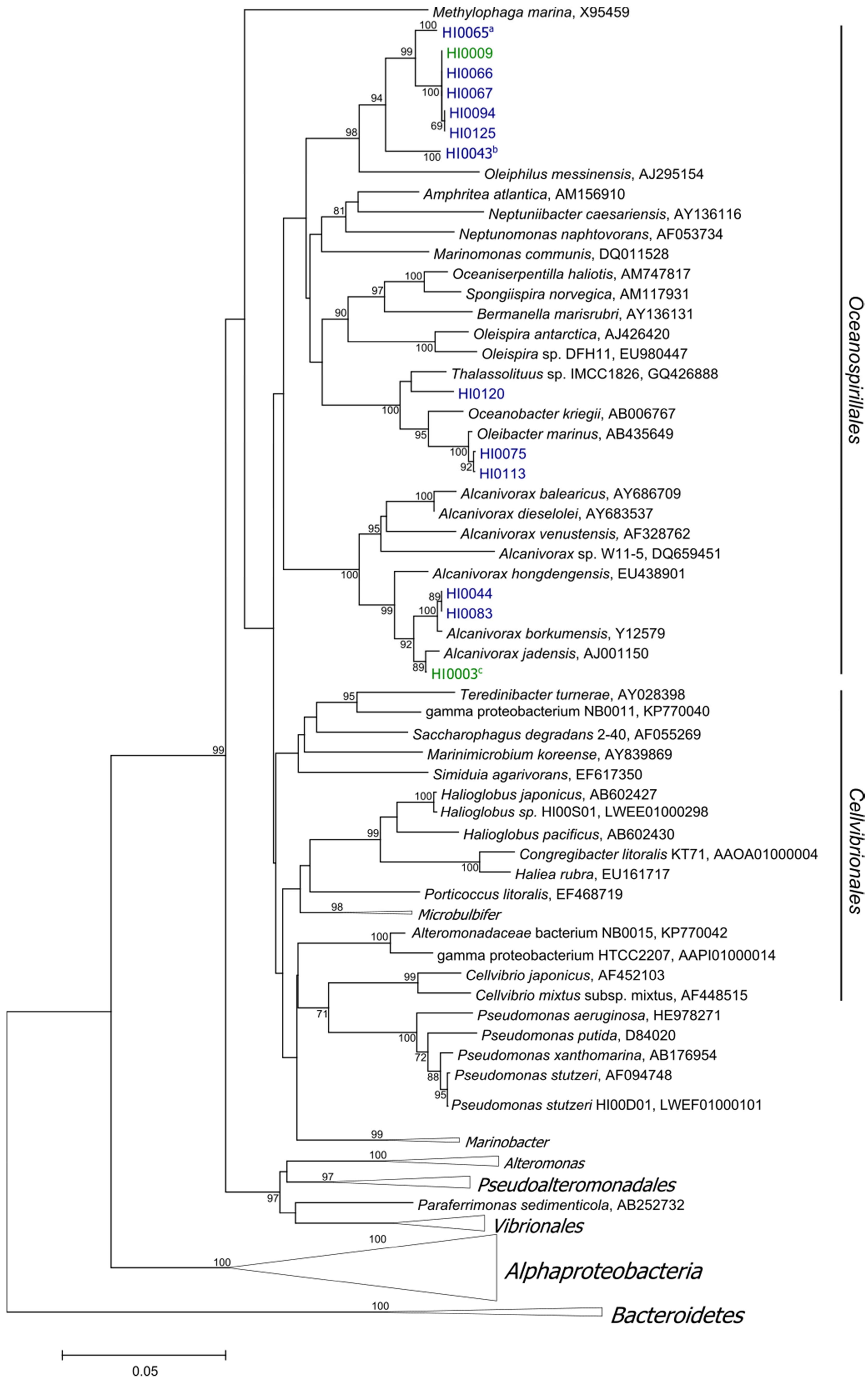

Supplementary Figure 1. SSU rRNA gene phylogeny of dilution-to-extinction isolates belonging to the gammaproteobacteria *Alcanivorax* and *Oleiphilus* genera of the order *Oceanospirillales*. Nodes with >70% agreement (1000 replicates) are shown. The scale bar indicates substitutions per site. [a]Grouping of HI0065 and 14 closely related sequences. [b]Grouping of HI0043 and 17 closely related sequences. [c]Grouping of HI0003 and five closely related sequences from the DCM. Only sequences with length >1400 bps were included.

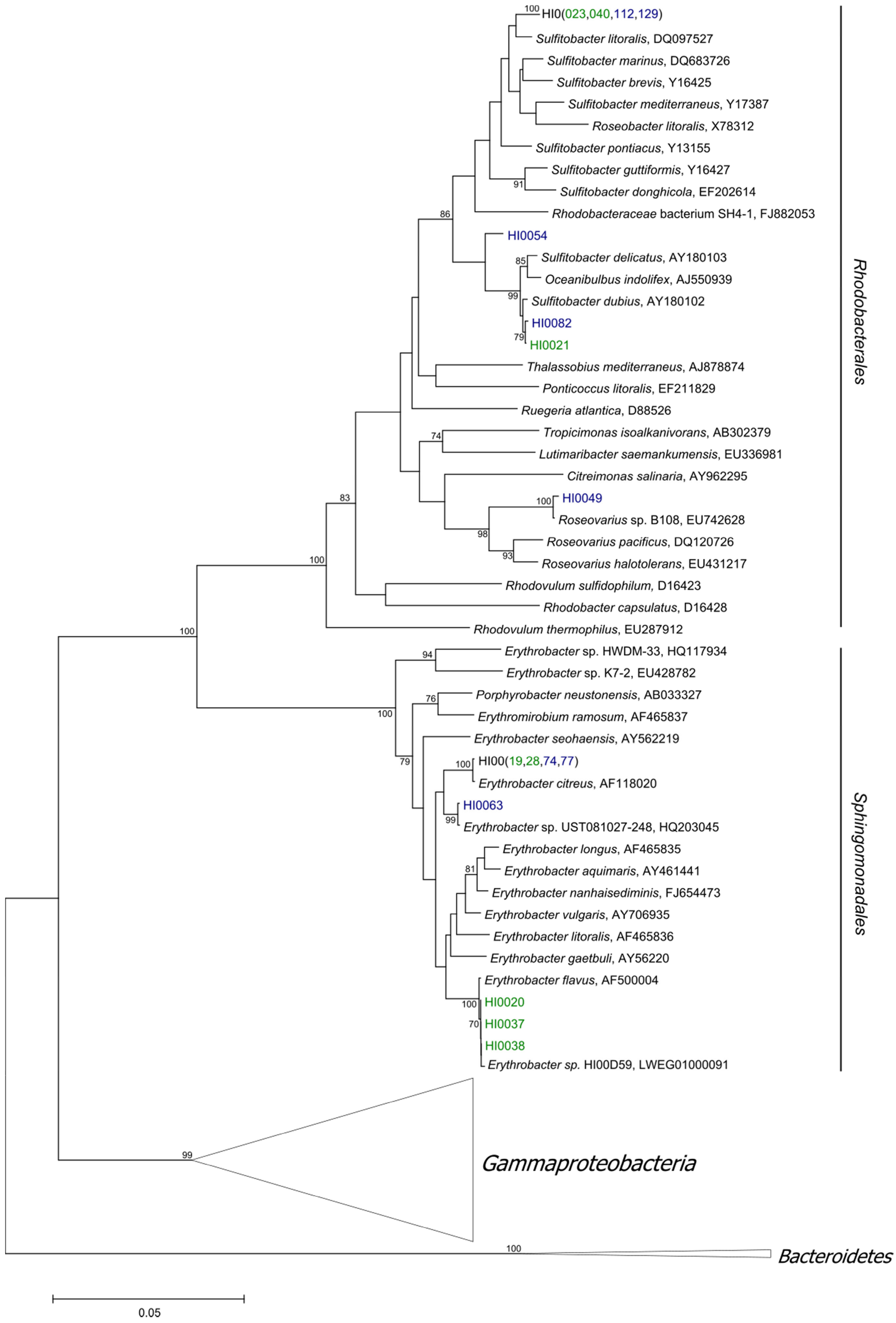

Supplementary Figure 2. The SSU rRNA gene phylogeny of dilution-to-extinction isolates belonging to the alphaproteobacteria orders *Rhodobacterales* and *Sphingomonadales*. Isolates marked in green were obtained from DCM samples. Isolates marked in blue were obtained from mesopelagic samples. Nodes with >70% agreement (1000 replicates) are shown. The scale bar indicates substitutions per site. Only sequences with length >1400 bps were included.

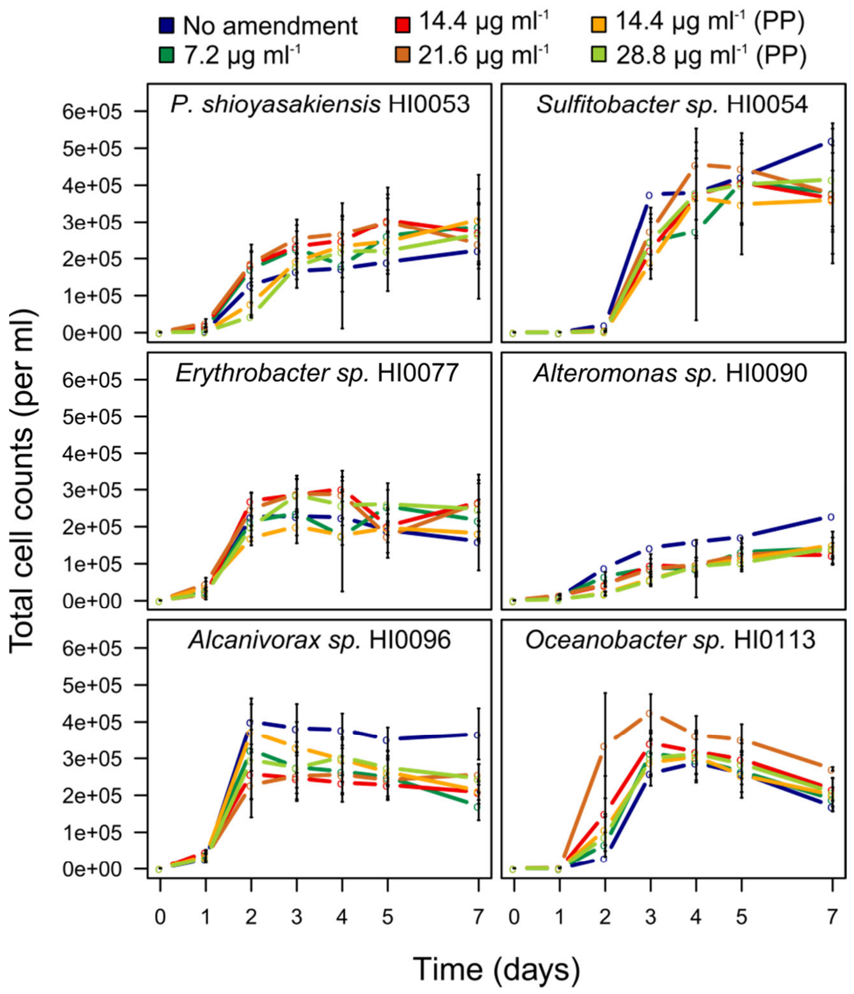

Supplementary Figure 3. Growth response of bacterial cultures supplemented with HMWDOM. The control treatment consisted of seawater medium without HMWDOM amendments. The final concentration of HMWDOM or purified HMWDOM polysaccharides (PP) each medium was amended with are indicated in the legend. The data indicates the mean cell count of triplicate samples at each time point. Errors represent the standard deviation.

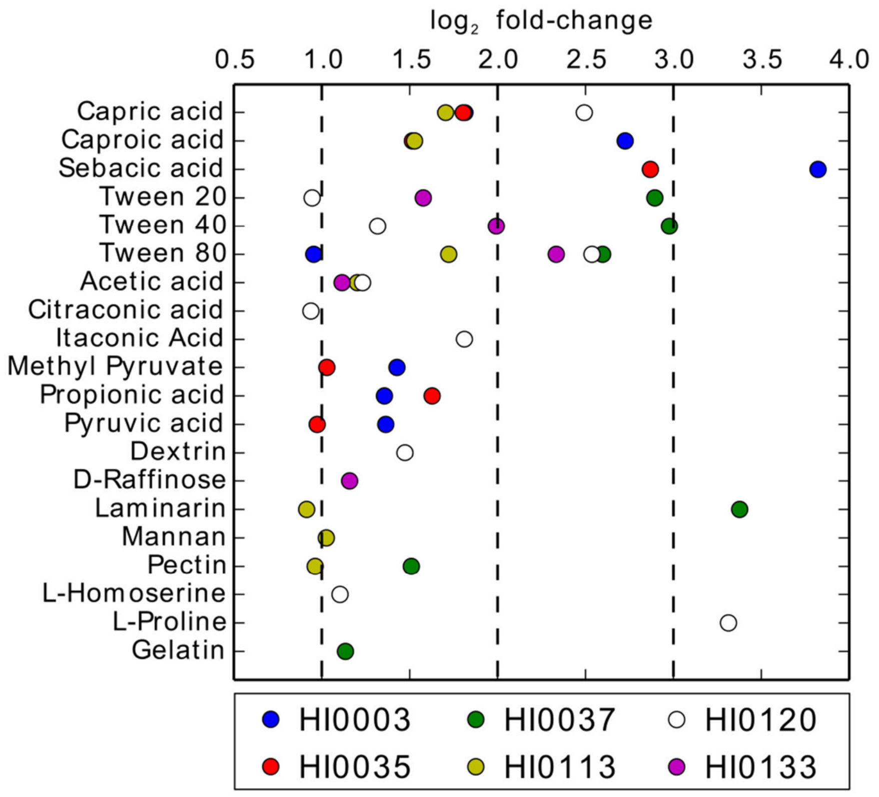

Supplementary Figure 4. Carbon substrate utilization profiles of hydrocarbon-degrading bacteria. Isolates tested included *Alcanivorax sp.* HI0003, *Alcanivorax sp.* HI0035, *Erythrobacter sp.* HI0037, *Oleibacter sp.* HI0113, *Thalassolituus sp.* HI00120, and *Oleiphilus sp.* HI0133. Biolog substrates for which the $\log_2$ fold-change was >0.9 were considered significant outliers and to supported positive growth. A $\log_2$ fold-change=2 indicates cell yields four times greater than the median cell yield for all substrates (N=190).

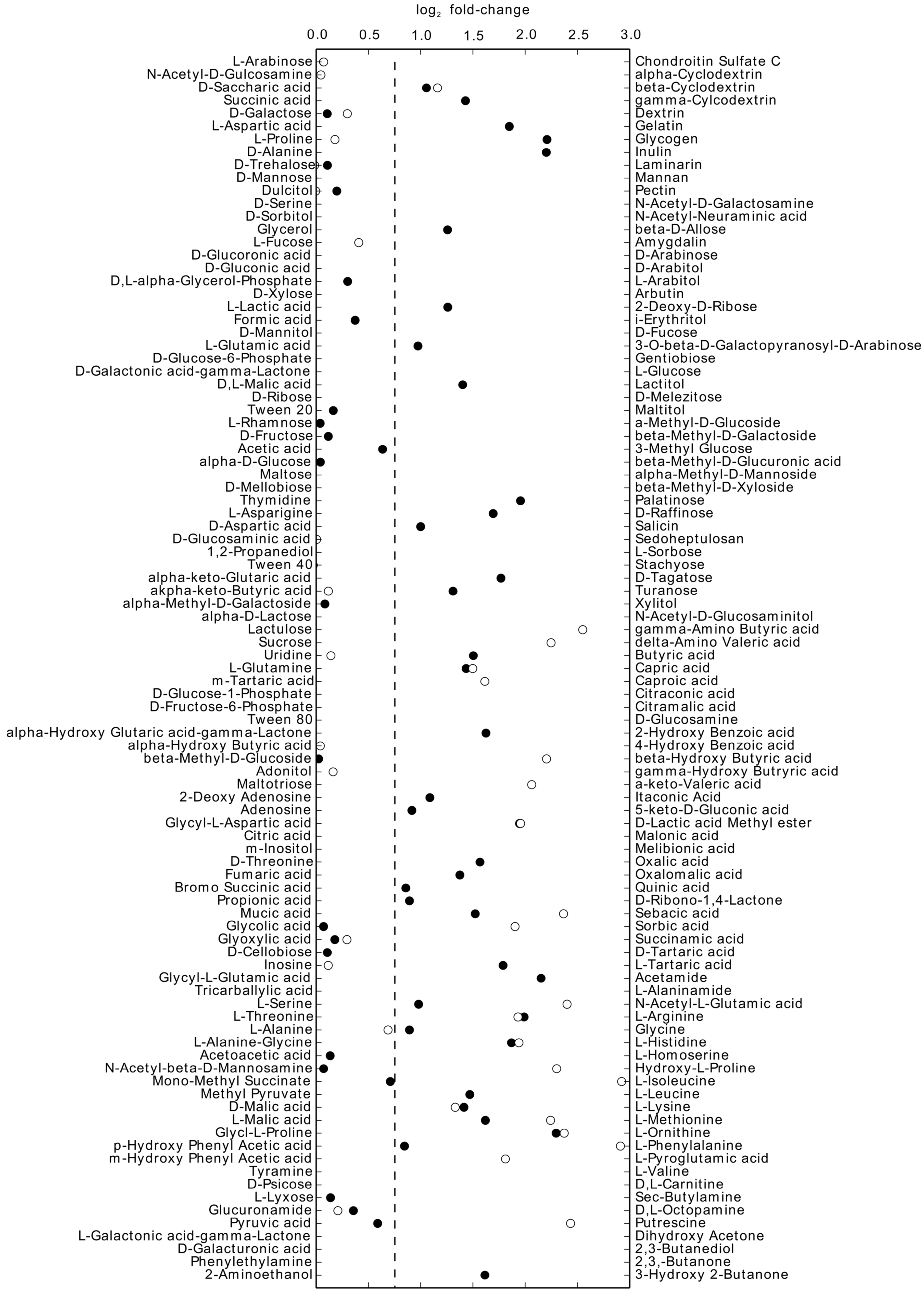

Supplementary Figure 5. Carbon substrate utilization profile of *Sulfitobacter sp.* HI0054. Biolog substrates for which the $\log_2$ fold-change >0.75 were considered as outliers and supported positive growth. A $\log_2$ fold-change=1 indicates cell yields two times greater than the median cell yield for all substrates (N=190). Filled circles correspond to test substrates indicated on the left and empty circles correspond to the substrates indicated on the right.

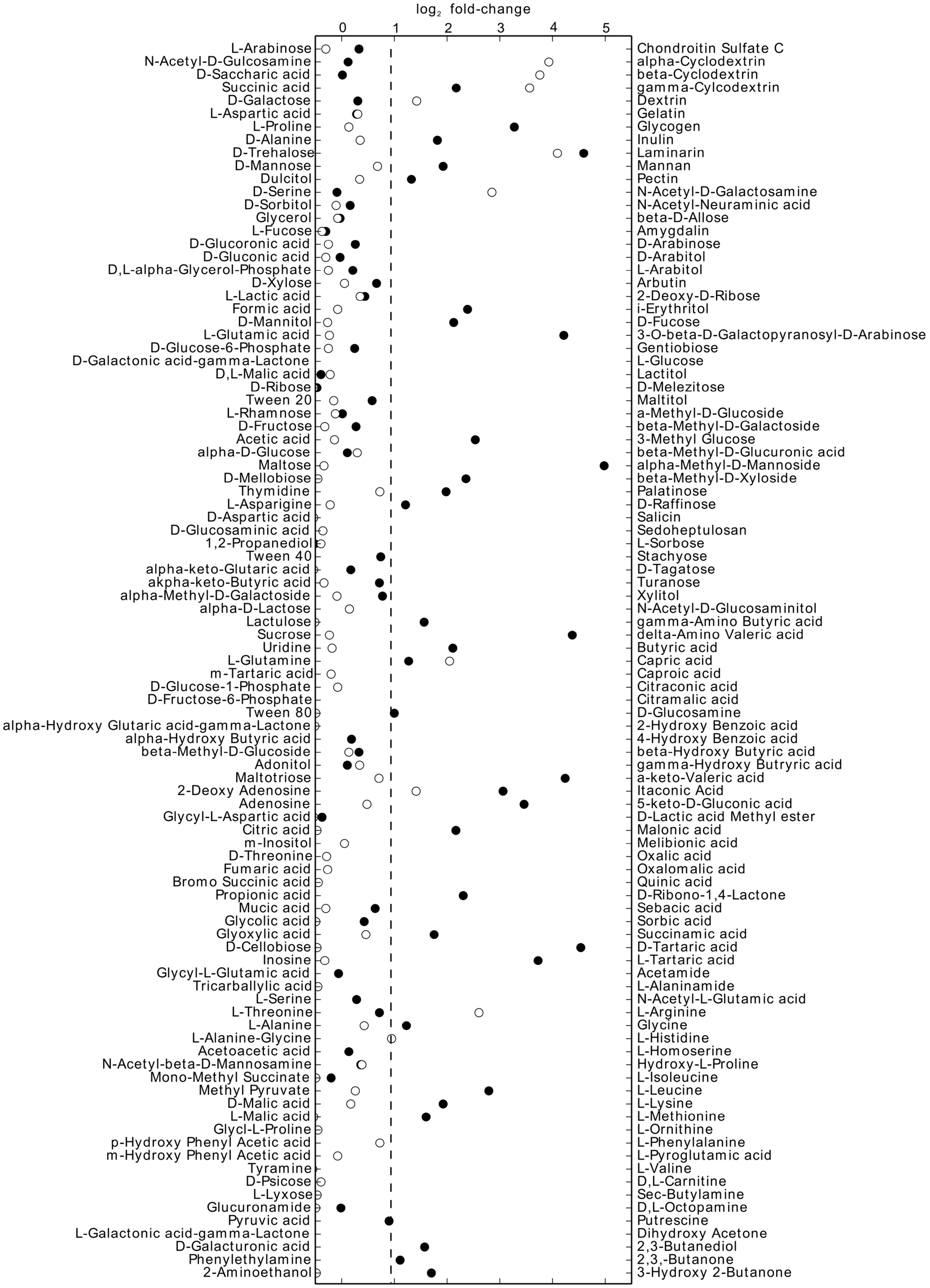

Supplementary Figure 6. Carbon substrate utilization profile of *Pseudoalteromonas shioyasakiensis* HI0053. Biolog substrates for which the $\log_2$ fold-change >0.94 were considered as outliers and support positive growth. A $\log_2$ fold-change=1 indicates cell yields two times greater than the median cell yield for all substrates (N=190). Filled circles correspond to test substrates indicated on the left and empty circles correspond to the substrates indicated on the right.
